# Experimental autoimmune encephalomyelitis in the common marmoset: a translationally relevant model for the cause and course of multiple sclerosis

**DOI:** 10.5194/pb-6-17-2019

**Published:** 2019-05-10

**Authors:** Bert A. 't Hart

**Affiliations:** 1 Department of Immunobiology, Biomedical Primate Research Centre, Rijswijk, the Netherlands; 2 Department of Biomedical Sciences of Cells and Systems, University Medical Center Groningen, the Netherlands

## Abstract

Aging Western societies are facing an increasing prevalence of chronic autoimmune-mediated inflammatory disorders (AIMIDs) for which treatments that are safe and effective are scarce. One of the main reasons for this situation is the lack of animal models, which accurately replicate clinical and pathological aspects of the human diseases. One important AIMID is the neuroinflammatory disease multiple sclerosis (MS), for which the mouse experimental autoimmune encephalomyelitis (EAE) model has been frequently used in preclinical research. Despite some successes, there is a long list of experimental treatments that have failed to reproduce promising effects observed in murine EAE models when they were tested in the clinic. This frustrating situation indicates a wide validity gap between mouse EAE and MS. This monography describes the development of an EAE model in nonhuman primates, which may help to bridge the gap.

## 1 Foreword

The marmoset experimental autoimmune encephalomyelitis (EAE) model was first documented in 1995 by the Florentine neurologist Dr. Luca Massacesi, who pioneered the model in the laboratory of Prof. Steve Hauser at UCSF (University of California, San Francisco, USA). During my research at the Biomedical Primate Research Centre (Rijswijk, the Netherlands) I had already some experience with an EAE model in rhesus monkeys (see review; Brok et al., 2001; 't Hart et al., 2005a), but was rather unhappy with the an acute clinical course of the model and the destructive neuropathology, which more closely resembled acute postinfectious demyelinating disease, such as acute disseminated encephalomyelitis (ADEM) than multiple sclerosis (MS; 't Hart et al., 2005a). The description of the new model in marmosets looked much better than our rhesus monkey EAE model and this became clearer once we started collecting our own data.

**Figure 1:**
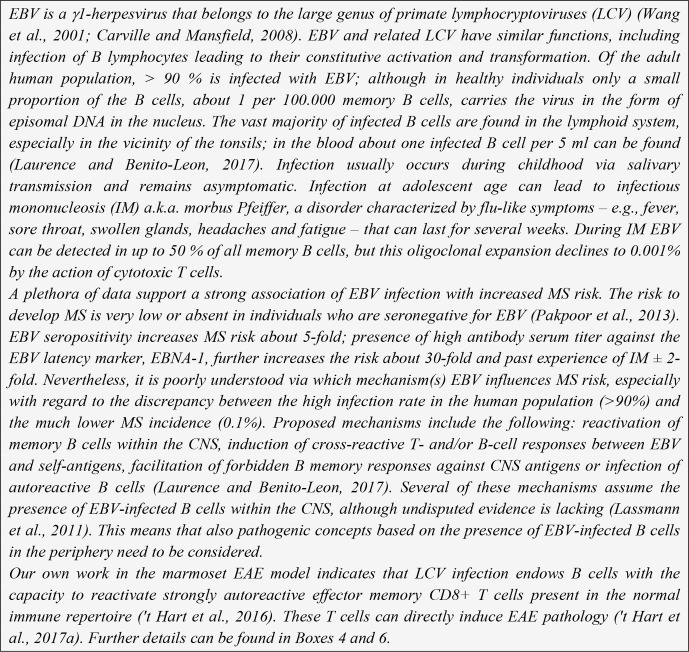
*Translational research: an iterative process.* The main goal of our exploratory preclinical research has been to find new targets in the pathogenic process for safer and more effective therapies. The translation of a new scientific discovery into a safe and effective innovative treatment for patients is indicated as forward translation. In the applied arm of our research, new therapies are tested. Results from such tests can be used to validate scientific concepts. When the process of forward translation fails, the reasons for failure should be investigated and this information should be fed back (i.e., reverse translation) to the animal model in order to make the necessary corrections in the scientific concept and/or the animal model itself.

An important success factor for the model has been our choice to focus on translational research into the pathogenesis as well as the treatment of MS. Our research thus stood on two legs, an exploratory leg where we unraveled (immuno)pathogenic mechanisms and an applied leg where the efficacy and safety of new therapies were tested (see Fig. 1). The underlying thought was that via this strategy we could use information from the applied leg to validate new pathogenic concepts developed in the exploratory leg.

At the start of our MS research in the marmoset EAE model, our thinking was strongly influenced by concepts developed in well-established EAE models in immunologically naïve rodents (i.e., specific pathogen-free, SPF, mice and rats). Indeed, we assumed that just like in the rodent models, autoreactive T cells in marmosets are naïve and require strong stimulation with danger signals for escaping regulatory mechanisms that keep them inactive (Matzinger, 1994). It took us many years to realize that this rather ignorant view was fundamentally wrong and that the immune systems of conventionally reared marmosets and SPF-bred mice or rats are for a large part incomparable. The same is true for the human immune system, as we can learn from the seminal work of Mark Davis and colleagues at Stanford University (CA, USA) (Davis, 2008; Brodin and Davis, 2017). There is now also mounting evidence that environmental factors have a profound influence on the immunocompetence of animal disease models. As an example, cohousing of SPF-bred laboratory mice with mice from pet shops or the wild creates a more human-like immune system (Beura et al., 2016). As stated by the authors of this hallmark publication, it is indeed ironic that an immunologically inexperienced 10–12-week-old mouse has become de rigueur for studies on the complex human immune system in health and disease (Beura et al., 2016).

We made the exciting discovery that EAE in marmosets is not driven by a single pathway but by two fundamentally different autoimmune pathways, one of which is more or less identical to the one in mouse EAE, while the other is completely different. The observation that the T cells that drive disease progression in the marmoset EAE model might be recruited from a repertoire of antigen-experienced T cells, which are absent in inexperienced SPF-bred rodents, underlies the concept that autoimmunity in MS might not be elicited by an infection (response-to-infection paradigm), but by primary injury inside the central nervous system (CNS; response-to-injury paradigm) ('t Hart et al., 2009).

**Figure 2:**
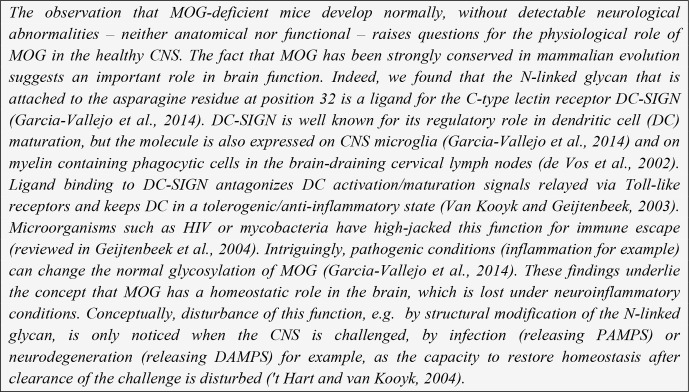
*Clinical and pathological aspects of marmoset EAE induced with MS myelin/CFA.***(a)** The graphs show the protracted clinical course, which is variable among individual animals. Case EH has relapsing–remitting disease that could be followed for almost 1 year. The other four cases transit to progressive disease, which can worsen quickly (EK, EL) or more slowly (EI, EJ). **(b)**  Case EI was subjected to in vivo magnetic resonance imaging (MRI) just before sacrifice. The middle and bottom rows show two horizontal brain slices from a T2-weighted image (middle) and a postcontrast (triple dose Gadolinium-DTPA; bottom) with the position of corresponding lesions indicated. After the scans were made, the monkey was humanely killed, the brain was removed and fixated in toto. Then a new postmortem T2-weighted scan was made. This allowed us to determine the exact position of all lesions that were detected in vivo. **(c)** The top row shows two coronal sections of the same MRI scan with lesions indicated. The middle and bottom rows show a magnification of individual lesions, which allowed us to conclude that brain lesions in this model are presented in different stages. **(d)** The top image shows an MRP14 staining of macrophages from lesions I and J, illustrating their inflammatory active nature. Notice that lesion I is one of the two gadolinium contrast-enhancing lesions. The three images below show the histological aspect of lesion J, which is characterized by primary demyelination (LFB staining), sparing of axons (Bielschowsky silver impregnation) and inflammation (MRP14). The macrophage staining shows the heterogeneity of this lesion, which is suggestive of confluent lesions of different age.

**Figure 3:**
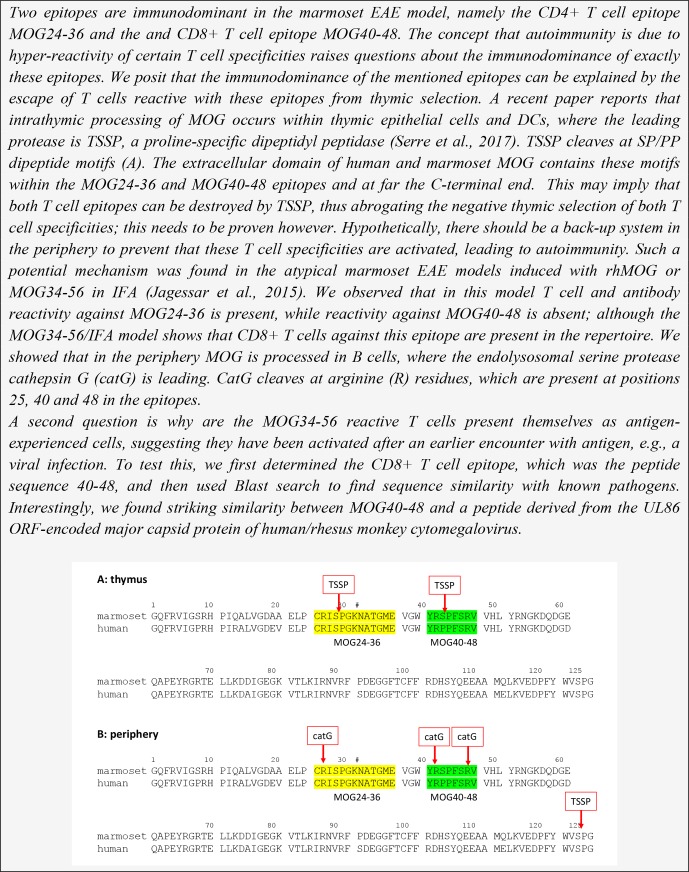
*Characteristic brain pathology of marmoset EAE induced with human MS myelin/CFA.* One brain hemisphere was immunostained for PLP to visualize demyelination and for MRP14 (macrophages/microglia) to visualize inflammation. For comparison, a brain hemisphere of a healthy marmoset was stained for PLP. Clearly visible is the different pathological aspect of white and cortical grey matter (cGM).

Our first experiments addressed basic questions on the diagnosis of disease symptoms and the visualization of CNS pathology in living animals. We designed a panel of MRI sequences that could be used for visualization and quantification of brain pathology in sedated EAE marmosets. A picture of the equipment can be found in the book *The Laboratory Primate* ('t Hart et al., 2005d). That work brought us a nice multidisciplinary publication, in which MRI, neuropathology and immunology were integrated ('t Hart et al., 1998). Figure 2 shows a compilation of figures from this publication and illustrates the strategy that we followed for obtaining histological information on MRI-detectable abnormalities in the EAE-affected marmoset brain. Particularly stimulating was the support from MS neuropathologists, who appreciated the remarkable neuropathological similarity of the model with MS. In retrospect, it is regrettable that we restricted our neuropathology examination in the first paper to the white matter. If we would have performed immunostaining for proteolipid protein (PLP), we might have been the first to report on the dramatic demyelination of cortical grey matter, a now very important pathological feature of MS that was then unknown (see Fig. 3).

The questions for subsequent experiments unfolded more or less logically:

- Which immunopathogenic mechanisms are operational in the periphery and within the lesions?
- What is the fine specificity of the T cells and antibodies that mediate the autoimmune attack on the CNS?
- Can the EAE induction protocol be refined by eliminating the role of nonspecifically acting factors, such as components of the adjuvant?
- Can methods be developed for longitudinal monitoring of the (immuno)pathogenic process?
- Can the MRI protocol be refined to get more information on lesion development in the intact animal?

The only research groups performing immunological research on marmosets were Prof. Claude Genain's group at UCSF (who stopped in 2007) and our group at the Biomedical Primate Research Centre (BPRC). Hence, we needed to work hard on the filling of our research toolbox with reagents (monoclonal antibodies, cytokine assays, PCR probes and primers) that could be used for immune profiling of our marmoset EAE model. We also had to develop assays suitable for the availability of only 3 mL venous blood per month. An overview of the currently used tools has been published (Jagessar et al., 2013b). The stream of publications in the peer-reviewed literature summarized in this monography shows that we have achieved most of our goals.

The question of whether there is a future for the marmoset EAE model has to be asked in these days, where biomedical research using live animals, primates in particular, is heavily debated in Europe and beyond. Scientists using nonhuman primates for the study of serious human diseases need to deal with increasing political and societal pressure as well as shortage of funding, both creating huge challenges. Our only arguments in the fight are the results of our research, which cannot be obtained in models in lower species, nor in patients, nor in cell or organ cultures.

Our research in the marmoset EAE model has provided highly relevant information for better understanding of MS as a basis for more effective therapies. In the past 5 years we have even entered the terra incognita of progressive MS, for which there is no valid alternative animal model and no effective treatment. I sincerely hope that this monography will enhance the appreciation of the translational relevance of nonhuman primate disease models among our diverse stakeholders. In addition, it might provide useful information in the dialogue with sectors of the human population criticizing nonhuman primate usage in biomedical research.

## 2 Preface

Due to their close phylogenetic relationship with humans, nonhuman primates (NHPs) provide translationally relevant models of a variety of diseases that threaten the aging human population (Mansfield, 2003; Tardif et al., 2011; 't Hart et al., 2012). The high degree of biological similarity of NHPs and humans is reflected in the immune system, which according to most experts is a central driver of the autoimmune neurological disease multiple sclerosis (MS). The translational relevance of NHP disease models should always be weighed against the high costs and justified ethical concerns, i.e., whether the same information can be obtained in a lower-ranked model or without animals. On the other side of the scale is the interest of the MS patients, who are desperately waiting for an effective treatment for their disease. I posit here that MS models in NHPs are indispensable in preclinical MS research as they can provide novel insights into mechanisms operating in the initiation and especially the progression of the disease that cannot be obtained in any other MS animal model, nor in the patient. Obviously, NHPs will never replace corresponding experimental models in adolescent (8–12 weeks old) inbred/SPF laboratory mice, but they definitely add complementary translational relevance to these models.

This monography reviews insights that we have gained during 25 years (1995–2020) of research in a model of experimental autoimmune encephalomyelitis (EAE) in the common marmoset (*Callithrix jacchus*). In this publication only the highlights can be discussed. For the underlying hard data, the reader is referred to the original publications given in the reference list.

## 3 Introduction

Aging Western societies are facing a steadily increasing prevalence of autoimmune-mediated inflammatory disorders (AIMIDs), for which no effective treatments exist, such as MS and type I diabetes. Despite significantly increased investment in the development of innovative therapies, the rate of drug candidates that reproduce promising effects observed in animal models when they are tested in the clinic, remains disappointingly low. The reasons for the low efficiency rate in therapy development for AIMID are unforeseen toxicity and a lack of efficacy, indicating that the predictive value of animal models for the clinical success of a candidate treatment is insufficient (Kola and Landis, 2004). However, funding for research aiming at understanding the reasons why treatments developed via forward translation (from the laboratory to the clinic) fail is limited. This is an unfortunate situation, as much can be learned from reverse translation research (from the clinic back to the laboratory) and the new insights can be used to improve currently used animal models ('t Hart et al., 2014).

The subject of this monography is the EAE model, an experimentally induced autoimmune disease in (genetically) susceptible laboratory animals that is projected on MS. EAE is not only the most frequently used animal model in preclinical therapy research for MS but has also often been used by immunologists as a generic model for testing basic concepts of immune tolerance and autoimmunity (Hohlfeld and Wekerle, 2004; Gold et al., 2006). However, mounting evidence indicates a wide immunological gap between frequently used EAE models in adolescent (8–12 weeks of age) mice or rats, which are bred and housed under very clean (SPF) conditions, and the MS patient. The important influence of the environment is elegantly illustrated by cohousing of SPF-bred mice with dirty mice, e.g., those purchased from the pet shop, which gives the SPF mice a more human-like immune system (Beura et al., 2016). The decisive influence of gut microbiota on EAE susceptibility is also illustrated in studies by Berer et al., who showed that EAE-prone transgenic mice, which do not develop evident disease under germ-free conditions, become spontaneously sick after administration of normal commensal gut microbiota (Berer et al., 2011). These and other studies underline the strong influence that gut microbiota can have on the competence of the murine immune system.

The fact that nonhuman primates are bred and raised under conventional conditions implies that, similar to the situation in humans, their immune system has been trained from early life onwards by genetic diversity and the exposure to environmental pathogens, gut microbiota as well as to chronic latent infection with herpes- and polyomavirus. Of note is that the gut microbiota of marmosets is highly enriched with bifidobacteria, which most likely is an adaptation to their specific dietary habits (e.g., gum) (Kap et al., 2018b); it most closely resembles the microbiome of human neonates (own unpublished observation). The fact that marmosets are naturally infected with β- and γ-herpesviruses is also particularly important; these viruses closely resemble those infecting humans (Nigida et al., 1979; Rivailler et al., 2002). It has been well established that endogenous and exogenous microbiota have a profound influence on the human immune system, as can be seen in aged people for example (Vallejo et al., 2004; 't Hart et al., 2013; Goronzy and Weyand, 2013; Vanheusden et al., 2015).

In summary, the fact that in nonhuman primates, autoimmune reactions develop within a pathogen-trained immune context marks an important difference with the situation in immunologically naïve inbred/SPF mice.

## 4 MS in a nutshell

**Figure 4:**
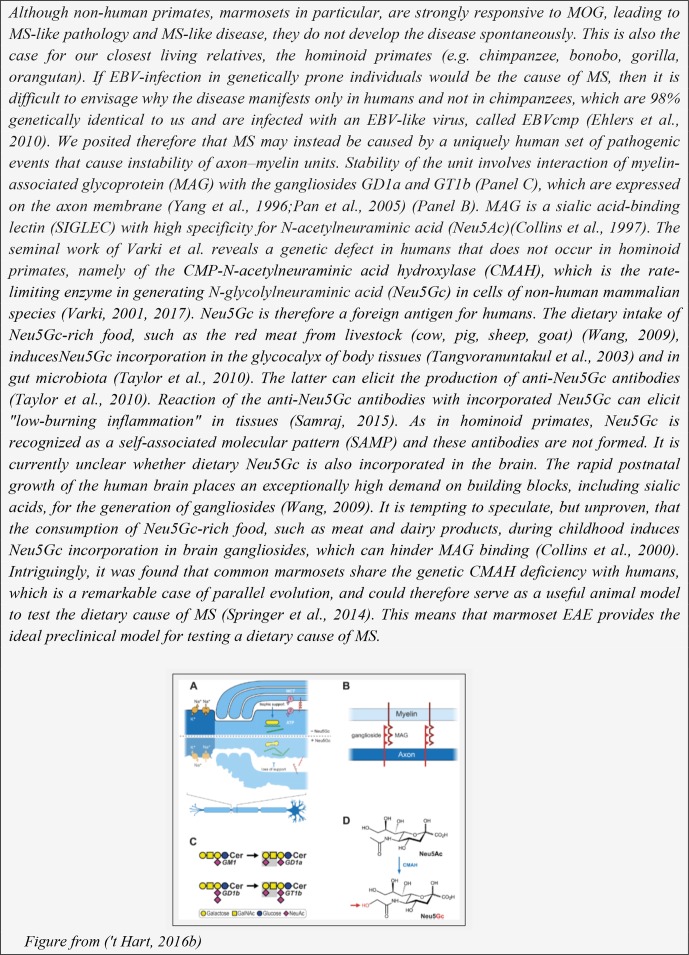
*Clinical aspects of multiple sclerosis.* **(a)** For quantification of the degree of disability, for example in the clinical assessment of new treatments, the expanded disability status scale (EDSS) has been developed (Kurtzke, 1983). **(b)** A graphical representation of the most common MS phenotype. In the beginning MS is asymptomatic, but with advanced imaging techniques (contrast-enhanced MRI for example) focal abnormalities due to inflammation can be observed inside the brain white matter. This is followed by a period of variable length between patients where the inflammation increases in severity causing discrete episodes of disability (relapse) alternating with complete recovery (remission). In about 50 % of the patients with relapsing–remitting MS the disease becomes progressive, where remissions disappear and neurological functions decrease progressively. Based on data from the marmoset EAE model, we posit that the degeneration of oligodendrocyte/myelin complexes can be differentiated into three types: (1) normal age-appropriate progressive degeneration; (2) progressive degeneration amplified by a newly discovered T cell attack on oligodendrocytes; (3) reversible degeneration induced by a classical autoimmune attack of pro-inflammatory T cells and autoantibodies on myelin sheaths.

MS is a chronic progressive neurological disease that specifically affects the central nervous system (CNS), which comprises the brain and spinal cord. Clinically, MS is characterized by increasing defects of sensory, motoric and/or cognitive functions. For quantification of the disease severity, symptoms are ranked on an expanded disability scoring scale (EDSS) (Fig. 4a). The EDSS is commonly used in clinical trials for quantifying the effect of a new treatment on the disease (Hohlfeld and Wekerle, 2004; Compston and Coles, 2008).

Conceptually, the disease course, as seen in the majority of MS patients (±85 %), can be divided into three phases that vary in length between individual patients (Fig. 4b). In the early, pre-symptomatic phase abnormalities can be detected in the brain white matter with the help of sophisticated imaging techniques, such as magnetic resonance imaging (MRI), but these pathological features are usually not expressed clinically. The subsequent phase, which can last for 5 to 20 years, is characterized by alternating episodes of neurological defects (relapse) and recovery (remission). This relapsing–remitting (RR) phase is followed by a secondary progressive (SP) phase, where symptoms worsen progressively and remissions disappear gradually. Note, there is no sharp separation between the RR and SP phases, and transition from RRMS to SPMS is usually assessed retrospectively (Lublin et al., 2014). In a minority of the patients (±15 %) the disease is progressive from the onset; this disease type is indicated as primary progressive MS (PPMS).

Based on evidence obtained in nonhuman primate EAE models, which will be discussed in more detail later, we posit that full development of disease activity in MS is caused by at least two distinct (and possibly sequential) pathological processes. We identified a classical combination of humoral and cellular autoimmune reactions, which induce episodic loss of neurological functions (relapses). In addition, we identified a novel cytopathic mechanism that seems to amplify the normal age-associated degeneration of myelin/oligodendrocyte complexes (Fig. 4b).

The exact nature of the events occurring at the transition from pre-symptomatic to relapsing–remitting MS (transition 1) and from relapsing–remitting to secondary progressive MS (transition 2) is debated. The simplest, albeit not necessarily correct, explanation may be as proposed in Fig. 4b, namely that transition 1 occurs when the mounting inflammatory pathology caused by the autoimmune attack exceeds a clinical threshold, whereas transition 2 occurs when the pathogenic influence of the immune system declines and progressive loss of function by irreversible degeneration of neuro-axonal and oligodendrocyte–myelin complexes becomes dominant. It is also proposed that in MS the age-related degeneration of oligodendrocyte–myelin complexes is enhanced by the pathological process. As was already mentioned and will be further discussed later, data from the marmoset EAE model revealed that this amplified degeneration may be mediated by a newly discovered pathogenic mechanism resembling neurodegenerative diseases (Alzheimer, Parkinson) (Bartzokis, 2004).

The pathological hallmark of MS and the most likely cause of the accumulating neurological deficits is the lesion, a usually well-defined area in the CNS white matter (WM), where the insulating myelin layers around axons are destroyed, but myelin-forming oligodendrocytes are spared. Depending on their age, activity and localization, lesions contain variable degrees of inflammation, repair (remyelination), astrogliosis (scar formation) and neuro-axonal pathology (Frohman et al., 2006). The presence of immune cells and molecules (antibodies, complement) in inflammatory active lesions, which are usually characterized by enhanced permeability of the blood–brain barrier (BBB), indicates involvement of the immune system in lesion formation. While MS was initially considered to be a typical autoimmune disease of the WM, it is now clear that the grey matter (GM) is affected as well, maybe even to a greater extent than WM (Geurts and Barkhof, 2008). In progressive MS the extent of cortical GM (cGM) demyelination, which can be focal in leukocortical and intracortical lesions or widespread in subpial lesions, often exceeds the load of WM lesions. Lesions in the cGM are often paucicellular, except in the very beginning of the disease (Lucchinetti et al., 2011), with only a rim of activated microglia cells. It is therefore suspected that demyelination of white and grey matter is caused by different pathogenic mechanisms. This view is supported by data from the marmoset EAE model, as will be discussed later in this monography.

In debates on the start of MS the terms cause and trigger are often used as synonyms, but I believe that these terms indicate clearly different entities. I like to use the First World War (WW1) as a metaphor to explain the difference. The cause of WW1 was a complex set of factors, including mounting economic problems in European countries and the increasing militarization of Imperial Germany. The trigger of WW1, however, was a single factor, namely the assassination of Archduke Franz Ferdinand of Austria and his wife Sophie by the Bosnian-Serb Gavrilo Princip. Whether the assassination would have sparked such a tragedy if the economic and social situation in Europe were more stable remains an open question.

Mutatis mutandis, the same may hold true for MS. While an undisputed trigger of MS has not been identified thus far, the disease risk profile indicates that there is a variety of causal factors that influence MS susceptibility; these can be genetic or environmental (Ascherio et al., 2012). Genomewide association studies (GWASs) reveal that the vast majority of genetic risk factors have a function in the immune system, although the individual contribution of each gene to the pathogenic process is often not clear. Environmental risk factors are diverse and include exposure to infectious (with Epstein–Barr virus) as well as noninfectious factors (smoking, sunshine exposure) (Ascherio and Munger, 2007; Ascherio et al., 2012). In the course of our research the question of whether MS might be triggered by an endogenous factor or process emerged.

Our work in the marmoset EAE model has been mainly focused on investigating the immunobiology of these MS risk factors, as potential causes of MS. The underlying thought was that via the analysis of MS risk factors insight could be gained into critical steps in the disease process.

## 5 MS and EAE: different or overlapping pathologies?

**Figure 5:**
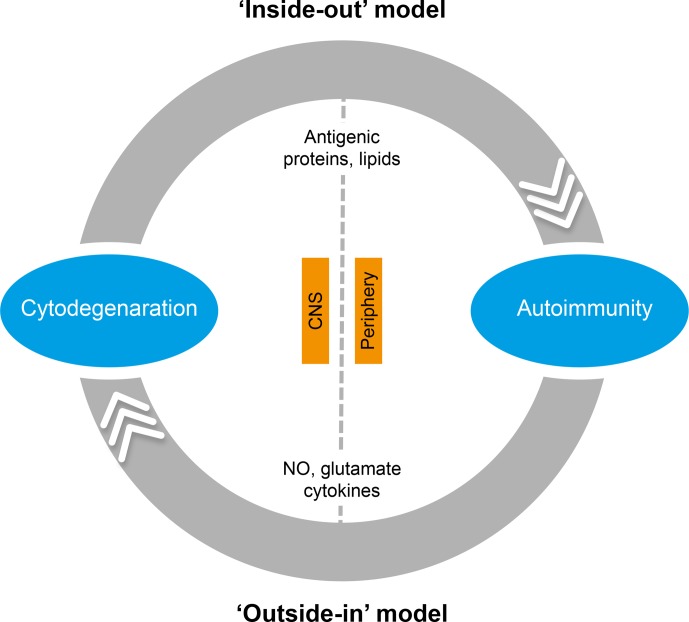
*Two opposing paradigms explaining the cause of autoimmunity in MS.* The prevailing concept is the outside-in paradigm, namely that infection of individuals who are genetically predisposed to MS with an as yet unidentified microorganism activates autoreactive T and B cells present in the normal immune repertoire. The autoimmune attack on the CNS induces cytodegeneration. Less commonly accepted is the inside-out paradigm, which states that a pathogenic event inside the CNS elicits the release of myelin antigens that activate autoreactive T and B cells present in the normal immune repertoire. The principle difference between both paradigms is that in the outside-in paradigm infection is the direct trigger of autoimmunity, whereas in the inside-out paradigm infections create a higher responsive state of the immune system.

In the literature, two essentially complementary etiological concepts for MS are presented (Fig. 5) (Stys et al., 2012). According to an outside-in paradigm, autoimmunity in MS is triggered in genetically predisposed individuals by infection with an exogenous pathogen; various mechanisms have been proposed, such as molecular mimicry and bystander activation (Fujinami et al., 2006). An inside-out paradigm proposes that MS starts as a degenerative process inside CNS myelin, indicated here as a primary lesion. Conceptually, the immune system reacts against antigens released from this primary lesion. It must be emphasized here that both concepts are based on dynamic interactions of genetic and environmental factors. However, while in the outside-in paradigm pathogens are the direct trigger of the autoimmune process, in the inside-out concept gene–pathogen interactions are a cause of MS, but not a trigger. Instead, they set the immune system in a hyper-responsive state to injury, which is triggered by another event ('t Hart et al., 2009). We will discuss later that data obtained in the marmoset EAE model are strongly supportive for the inside-out paradigm.

The inside-out paradigm harmonizes nicely with a much older “primary lesion” theory of Terence Wilkin (Wilkin, 1990). This theory essentially implies that

- autoimmunity is not itself an entity, but a physiological response to sustained excess antigen turnover in diseased tissues (the primary lesion).
- those who develop clinical disease are viewed as high responders to critical antigens.

The first proposition implicates a mechanism that amplifies the normal age-associated degeneration of myelin-oligodendrocyte and neuro-axonal complexes (as postulated in Fig. 4). As will be discussed later, such a mechanism has been found in the marmoset EAE model. The second proposition implies a hyper-immune response of MS patients against myelin components compared to healthy individuals. This has been shown by several authors (Kerlero de Rosbo et al., 1993; Bielekova et al., 2004). As will be discussed later, we found a dominant antigenic role of the quantitatively minor constituent myelin oligodendrocyte glycoprotein (MOG) in the marmoset EAE model.

**Box 1:**
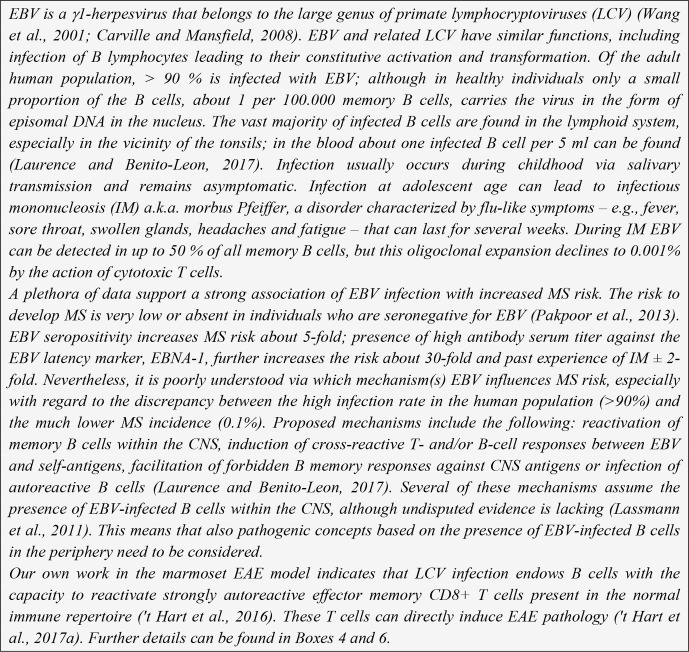
Epstein–Barr virus (EBV).

It is heavily debated whether studies in an EAE model can provide relevant insights into the mechanisms that trigger and/or perpetuate MS (Sriram and Steiner, 2005; Steinman and Zamvil, 2005; 't Hart et al., 2011; Ransohoff, 2006). The essential difference between MS and EAE is that MS develops spontaneously in (genetically) predisposed individuals, while EAE is experimentally induced in otherwise healthy animals by means of rather artificial procedures (Laman et al., 2017). Historical data indicate, however, that there may be no sharp separation between the two diseases. Humans were found to be susceptible to EAE. In fact, the EAE model stems from the observation by Pasteur that humans vaccinated with inactivated rabies virus grown on rabbit brains, developed brain pathology reminiscent of an acute demyelinating disease in humans, called acute disseminated encephalomyelitis (ADEM) (Pasteur, 1885). Subsequent work by Rivers et al. (1933), Rivers and Schwenkter (1935) and Kabat et al. (1947) in rhesus macaques clarified that this neurological disease was not caused by the virus, but induced by components from the rabbit brain that contaminated the vaccine. Collectively, the published data imply that sensitization of humans and nonhuman primates (i.c., rhesus monkeys) against antigens present in the rabbit brain elicits similar ADEM-like pathology and disease. On the other hand, there is a report from the Oregon National Primate Research Center describing cases of spontaneous MS-like disease in a colony of Japanese snow monkeys (*Macaca fuscata*). The disease could be attributed to infection with a new γ2-herpesvirus that is most closely related to a rhesus macaque rhadinovirus (Axthelm et al., 2011). Interestingly, also in the mouse a rhadinovirus, murine gammaherpesvirus-86, has been implicated as a trigger of MS-like disease (Marquez and Horwitz, 2015). It remains an intriguing question why MS-like disease in animals is associated with a γ2-herpesvirus, while the human disease is associated with a γ1-herpesvirus, the lymphocryptovirus (LCV) EBV (Box 1).

To our knowledge there is no clear evidence that γ2-herpesviruses, Kaposi sarcoma herpesvirus (KSHV) for example, have a pathogenic role in MS. Although both γ1- and γ2-herpesviruses infect neuronal cells as well as B cells in vitro, such as in Hodgkin's and primary effusion lymphoma, respectively (Jha et al., 2015), it is unclear at this stage whether γ2-herpesviruses activate cellular pathways relevant to T-cell-mediated autoimmune disease, such as those described recently for EBV-infected B cells (peptide citrullination and activation of the autophagy pathway) ('t Hart et al., 2016). The infected snow monkeys display MS-like pathology in the white matter of the cerebellum as well as oligoclonal bands in their cerebrospinal fluid (CSF) (Blair et al., 2016). However, the paper does not explicitly mention that demyelination was detected in cortical grey matter (cGM), which might mean that this pathological hallmark of MS is absent. Our own data in the marmoset EAE model (reviewed in the following paragraphs) indicate that cGM and WM pathology may be distinct entities induced via different pathogenic mechanisms (see below) and that the γ1-herpesvirus is implicated in the pathway leading to GM pathology ('t Hart et al., 2017a).

## 6 Nonhuman primate models of EAE

Rivers and Schwenkter (1935) reported that macaques given repeated (60 or more) intramuscular injections of alcohol-ether extracts from normal rabbit brain developed similar ADEM-like pathology as observed in earlier studies (Rivers and Schwenkter, 1935). Subsequently, Kabat et al. (1947) observed that ADEM-like disease could also be induced with only a few injections (∼3) of heterologous (rabbit) or even autologous (rhesus monkey) brain extracts, provided that the extracts were mixed with paraffin oil in which heat-killed dried tubercle bacteria were suspended (Kabat et al., 1947). This powerful immune-potentiating paraffin oil/mycobacteria mixture is nowadays commercially available as complete Freund's adjuvant (CFA) and is the most frequently used, albeit disputed, adjuvant in EAE studies (Laman et al., 2017).

The good thing of CFA is that it elicits both cellular and humoral autoimmune reactions against coformulated antigens. The bad thing is that the adjuvant skews the differentiation of antigen-activated CD4+ T cells towards a pro-inflammatory profile (Billiau and Matthys, 2001), which often does not necessarily reflect the unbiased adaptive immune response of the inoculated animal. Moreover, the injection of CFA into the skin provokes ulcerative lesions at the injection sites, which causes serious discomfort to the animals. Recently we reported that robust clinical EAE could be induced in three nonhuman primate species – rhesus monkey, cynomolgus monkey and common marmoset – with the myelin antigen myelin oligodendrocyte glycoprotein (MOG) formulated with only the oil without mycobacteria (incomplete Freund's adjuvant; IFA) (Haanstra et al., 2013b). This discovery not only implies a major reduction of discomfort for the animals (refinement), but also enables interrogation of the animal's immune repertoire for the presence of pathogenic cells without the need of aspecific co-stimulation.

The EAE research at the Netherland's primate center (in 1994 renamed to BPRC) was initiated by Margreet Jonker, who set up the model in rhesus monkeys (van Lambalgen and Jonker, 1987b, a; Jonker et al., 1991). Observations by Rose et al. (1994) could be replicated, namely that intracutaneous injection with myelin or with the quantitatively major myelin basic protein (MBP) formulated with CFA elicited an acute neurological disease with serious inflammatory/necrotic lesions. Disease development in this model was associated with increased numbers of neutrophilic granulocytes in the blood as well as in the lesions. Interestingly, the same formulations elicited EAE with only mild inflammatory lesions in marmosets (Brok et al., 2000). The beneficial effect of anti-CD4 monoclonal antibody in the rhesus monkey EAE model indicates that there must be an underlying adaptive autoimmune process (van Lambalgen and Jonker, 1987b, a), which may be aggravated by innate immune reactions against components of the adjuvant that cause the serious damage. Recent work by Dunham et al. (2017b) shows that the combination of intra-CNS oxyradical production by infiltrated neutrophils and redistribution of iron generates toxic oxygen ($O_{2}^{-}$, $H_{2}O_{2}$) and nitrogen (NO) species that can seriously aggravate tissue destruction in the lesions (Dunham et al., 2017b). Note, the different severity of tissue destruction is reflected at the antigen/CFA inoculation sites in the skin; in rhesus monkeys serious granulomas are formed, while these are much less severe in marmosets. The beneficial effect of the neutrophil oxidative burst antagonist apocynin on the skin granuloma formation in rats demonstrates that neutrophils have a prominent role in tissue destruction ('t Hart et al., 1992).

Although the rhesus monkey EAE model has been useful for the efficacy testing of therapeutic agents, which due to insufficient cross-reactivity could not be tested in marmosets (Haanstra et al., 2013a, 2015), we believe that the EAE model in marmosets is more relevant for MS.

## 7 EAE in marmosets: a superior primate MS model

### 7.1 What is a marmoset?

**Figure 6:**
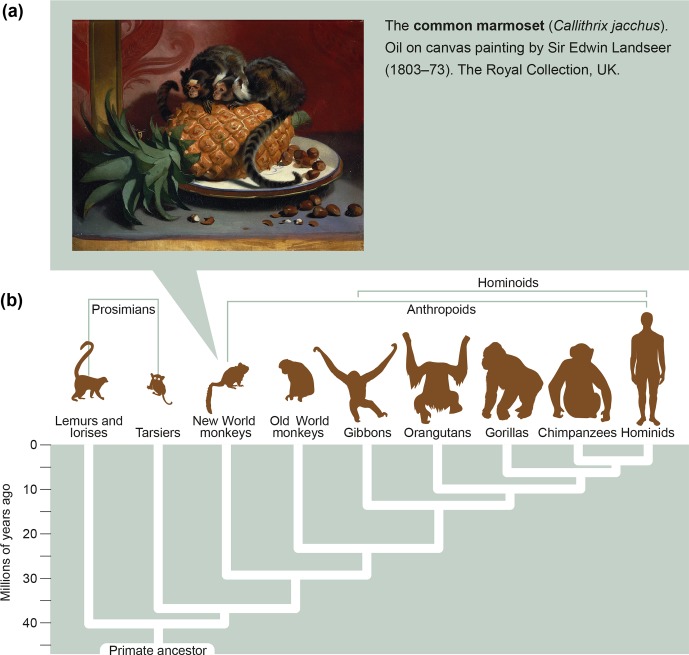
*What is a marmoset?* Depicted is an artist's impression of two marmosets (*Callithrix jacchus*) showing their small body size (oil on canvas painting by Sir Edwin Landseer, 1803–1873, the Royal Collection, United Kingdom). The lower part of the figure depicts the phylogenetic tree of primates with the evolutionary distance to humans.

Marmosets are small-bodied nonprotected primates, weighing 350–400 g at adult age, which have their natural habitat in the Amazon forest. Figure 6a shows the features and size of these animals. As a detailed description of the biology of marmosets and their usage in preclinical research is beyond the scope of this monography, we refer the reader to reviews published elsewhere (Haig, 1999; Mansfield, 2003; Ludlage and Mansfield, 2003; Tardif et al., 2003, 2011; 't Hart et al., 2012). To name a few important aspects, marmosets breed well in captivity, where they can give birth to two nonidentical twins per year. Due to the sharing of the placental bloodstream, the immune systems of fraternal siblings are educated in the same thymic compartment and are therefore very comparable. This creates an attractive experimental setting for two-leg studies comparing the effect of an experimental variable, a new therapy for example, with a control preparation.

**Box 2:**
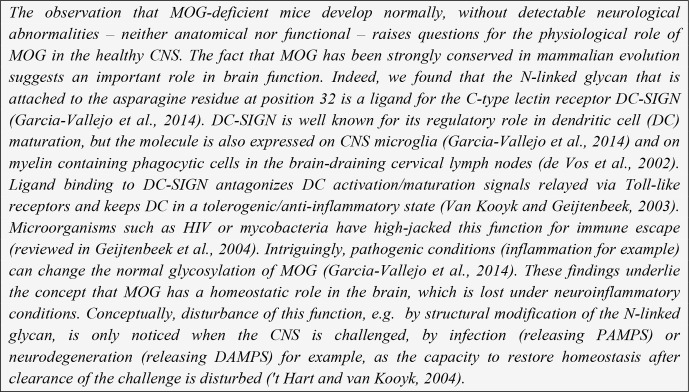
Dual role of MOG, as a tolerogen or immunogen.

The BPRC houses a large self-sustaining colony of marmosets, comprising between 100 and 150 animals. The monkeys are kept in family groups that are housed in spacious cages with indoor and outdoor enclosures. This means that our marmosets are exposed from birth to environmental microbes as well as to intestinal microbiota and chronic latent infections (EBV; cytomegalovirus, CMV) carried by family members. Evidence shows that these microorganisms have an important effect on the immune status; in other words marmosets have a pathogen-educated immune system. Mounting evidence indicates that this daily contact with bacteria and viruses from in- and outside the body generates immune functions that are complementary to the classical ones found in the inbred/SPF laboratory mice used in most immunological research.

### 7.2 Some immunological characteristics of the marmoset relevant to preclinical studies in the EAE model

- *MHC.* Genomewide association studies in MS and results obtained in rodent EAE models show that the highly polymorphic major histocompatibility complex (MHC) class II region exerts the strongest genetic influence on disease susceptibility (Sawcer et al., 2011, 2014). The main biological function of MHC class II molecules is to present antigens to CD4+ T cells, which dominate the immunopathology in mouse EAE models, but seem to have a less prominent role in MS (Lassmann and Ransohoff, 2004). Alleles constituting the HLA-DR2 haplotype, HLA-DRB1*1501/-DRB5*0101/-DQB1*0602, have the strongest risk association with MS in the Caucasian populations of northern Europe. Disease associations with the MHC class I region have been much less intensively investigated, which is remarkable as CD8+ T cells, which receive their antigens presented by MHC class I, are the dominant T-cell subtype in MS lesions.
  The MHC class II region of marmosets comprises the equivalents of HLA-DR and -DQ, which are respectively indicated with the acronyms Caja-DR and -DQ (from *Callithrix jacchus*); Caja-DP genes are absent (Antunes et al., 1998; Doxiadis et al., 2006). Three Caja-DR loci have been identified: two produce functional transcripts, namely the monomorphic Caja-DRB*W1201 and the polymorphic Caja-DRB*W16 loci, while the Caja-DRB1*03 locus contains only pseudogenes. Two hybrid Caja-DRB transcripts were found, which are composed of exon 1 and 3 of Caja-DRB*W16 alleles with exon 2 of Caja-DRB1*03 alleles. Furthermore, oligomorphic Caja-DQA1 (2 alleles), -DQB1 (3 alleles) and -DQB2 (2 alleles) genes were found. The invariant Caja-DRB*W1201 product is particularly relevant for the EAE model as it restricts the activation of CD4+ T cells specific for rhMOG epitope 24–36, which have a key function in the initiation of the disease (Brok et al., 2000).
  The MHC class I region of marmosets has been less well characterized (van der Wiel et al., 2013). Sequences comparable to the classical HLA-A, -B and -C genes have not been found. Evidence was found for functional transcripts of the oligomorphic Caja-E locus (2 alleles). Caja-E has an important role in the marmoset EAE model as it restricts the activation of autoaggressive cytotoxic T cells (CTL) specific for rhMOG epitope 40–48 (Jagessar et al., 2012d). The classical antigen presentation function to CD8+ T cells seems to be executed by products from the polymorphic Caja-G locus (van der Wiel et al., 2013).
- *TCR.* The repertoire of T cell antigen receptors (TCRs) has been unraveled (in part) (Uccelli et al., 1997). Overall a high similarity of marmoset and human TCRBV-D-J-C sequences was observed, illustrating the close phylogenetic relationship between the species.
- *BCR.* The repertoire of B cell antigen receptors (BCR) has been partly unraveled as well (von Budingen et al., 2001). Also at the level of the immunoglobulin heavy variable (IGHV) gene repertoire a high similarity was found between marmosets and humans.
- *Other relevant immune molecules.* The initiation and perpetuation of (auto)immune reactions involves interaction of various cell types via multiple cell-bound or secreted molecules. Some insight into the degree of relatedness between those molecules from humans and marmosets can be gained by testing whether monoclonal antibodies (mAbs) raised against human surface-expressed CD molecules cross-react with marmoset cells. It is obviously a major effort to do this for all immune molecules, but for several the data have been published and the list is periodically updated (Jagessar et al., 2013b).

In conclusion, the relatively close evolutionary distance of marmosets and humans, which has been estimated at about 30 million years (Fig. 6b), is reflected by a high degree of immunological similarity. This makes the marmoset an exquisitely attractive animal model of human AIMIDs.

### 7.3 EAE induction in marmosets

The first report on successful EAE induction in marmosets was published in 1995 (Massacesi et al., 1995). The authors used a rather harsh immunization protocol, involving human brain homogenate formulated with CFA containing 3 mg mL${}^{-1}$ *Mycobacterium tuberculosis*. In addition, they gave intravenous injections of 10${}^{10}$ inactivated *Bordetella pertussis* particles at the day of immunization and 2 d later. This resulted in a relapsing–remitting (RR) disease course with inflammatory–demyelinating pathology. The authors also showed that EAE could be transferred within (bone marrow chimeric) twins from an EAE-affected monkey to its fraternal sibling using MBP-specific T cell lines. In later studies by this group, which were led by the neurologist Dr. Claude Genain, several important findings were made, such as the detailed unraveling of the repertoire of immune reactions against MOG, the crucial role of anti-MOG antibodies for demyelination, the presence of anti-MOG antibodies inside lesions and the unexpected late exacerbation of neurological disease occurring after tolerization against MOG (reviewed in Genain and Hauser, 2001).

The harsh experimental procedures used by Genain and Hauser (2001) are a clear reflection of the prevailing SPF rodent-based concepts on EAE and MS in the 1990s, without taking into account that the immune systems from SPF rodents and non-SPF primates might differ fundamentally. Our group at the BPRC chose another approach, namely a stepwise refinement of the marmoset EAE model to the minimal components needed for the induction of MS-like pathology and disease. These efforts are summarized in the following.

Inspired by the seminal publication of Massacesi et al. (1995), we used the same EAE induction protocol in marmosets from our own purpose-bred colony ('t Hart et al., 1998). The only modification was that we used myelin isolated from an MS brain, which we purchased from the Netherlands Brain Bank (Amsterdam, the Netherlands). Similar to the original study, we obtained relapsing–remitting (RR) EAE with severe inflammatory pathology. However, we noticed that clinically evident EAE, albeit with a more protracted course and less destructive pathology, could be obtained when we used a commercially available CFA preparation (Difco) containing only 1 mg mL${}^{-1}$ mycobacteria and left out the Bordetella particle injections. The huge advantage of this modified protocol for the animals was that the inoculum provoked considerably less skin ulceration.

The observation by McFarland et al. (1999), that demyelination in marmosets immunized with a chimeric MBP/PLP protein in the adjuvant Titermax was always associated with spreading of the immune response to MOG, underscores the pathogenic relevance of this quantitatively minor CNS myelin component. Data summarized in Box 2 indicate that MOG is a remarkable molecule with a Dr. Jekyll/Mr. Hyde-type of role in the brain, which depends on its glycosylation state. We tested whether immunity to MOG is indeed essential for EAE development. Our approach was to immunize fraternal twin siblings with myelin from B6 mice, which were either wild-type or MOG-deficient mice. MOG-deficient mice had been developed by Delarasse et al. (2003). We observed that in one twin with acute onset EAE the absence or presence of MOG in the myelin inoculum made no difference for the EAE presentation, while in the other twin, development of chronic EAE was impaired when MOG was absent. At the pathological level, demyelination was reduced albeit not absent (surprisingly) in the sibling immunized with MOG-deficient myelin (Jagessar et al., 2008)!

**Figure 7:**
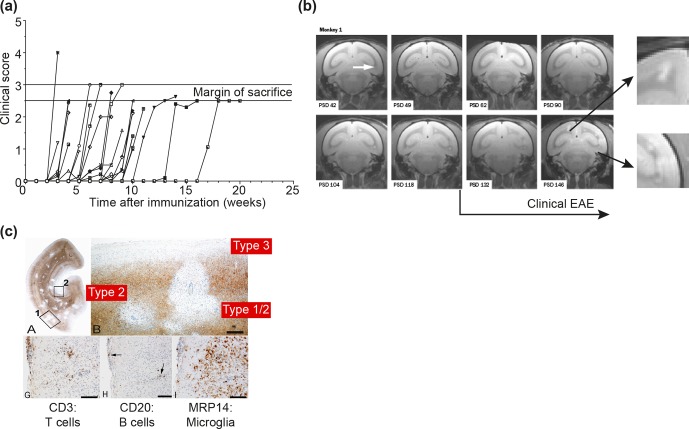
*Clinical and pathological aspects of the marmoset EAE model induced with rhMOG/CFA*. **(a)** Depicted is the EAE course in a representative selection of 30 unrelated marmosets receiving a single immunization with rhMOG/CFA on day 0. All monkeys developed clinically evident EAE, but the time of onset varied from 2 to 16 weeks. **(b)** Serial imaging of a case with late EAE onset, showing early onset of brain lesion formation. The white arrow points to the first detectable lesion. Clearly visible is that formation of new lesions in the depicted brain slice (0.5 mm) is disseminated in time and space. Around the time that clinical signs were diagnosed, lesion colonization of cortical grey matter is detectable (inserted magnifications). **(c)** PLP staining of a brain hemisphere shows the dramatic demyelination in white and grey matter **(a)**. Different grey matter lesion types identified in the MS brain can be distinguished **(b)**. Lesions are paucicellular with regard to T (CD3) and B (CD20) cells, but contain abundant MRP14+ myeloid cells, representing microglia and macrophages.

The previous findings show that autoimmunity against MOG is indispensable for the development of chronic EAE in marmosets. To further investigate the anticipated complex patterns of cellular and humoral autoimmunity against MOG in the genetically outbred marmoset EAE model, we immunized marmosets with a nonglycosylated recombinant protein expressed in *Escherichia coli* bacteria that represents the extracellular domain (residues 1–125). Possible epitopes located in the transmembrane and intracellular parts of the molecule, which were identified for mice and rats, were excluded from our studies ('t Hart et al., 2011). The protein was formulated with CFA and the emulsion was injected once at day 0. The clinical course pattern in a representative cohort of 30 monkeys immunized with rhMOG/CFA is depicted in Fig. 7a, showing that the disease incidence is 100 %; while in this collection of EAE cases the time of EAE onset varied from 2 to 16 weeks. Our original idea that this variation reflects the genetic heterogeneity of the monkeys proved to be incorrect as was shown by serial magnetic resonance brain imaging (MRI). In the depicted example (Fig. 7b), T2 lesion development was detectable already within a few weeks after immunization, while clinical signs were diagnosed many weeks later. It was noticed in this example that very late in the disease and around the time that clinical signs could be diagnosed, lesions started to “colonize” the grey matter of the cerebral cortex. We drew the tentative conclusion from these observations that the formation of CNS lesions and the induction of neurological deficits might be based on distinct immunopathogenic mechanisms (for details see also Box 3). Immunostaining of a cerebral hemisphere from an rhMOG/CFA-immunized monkey for PLP confirmed the presence of demyelination in WM and cGM (Fig. 7c).

**Box 3:**
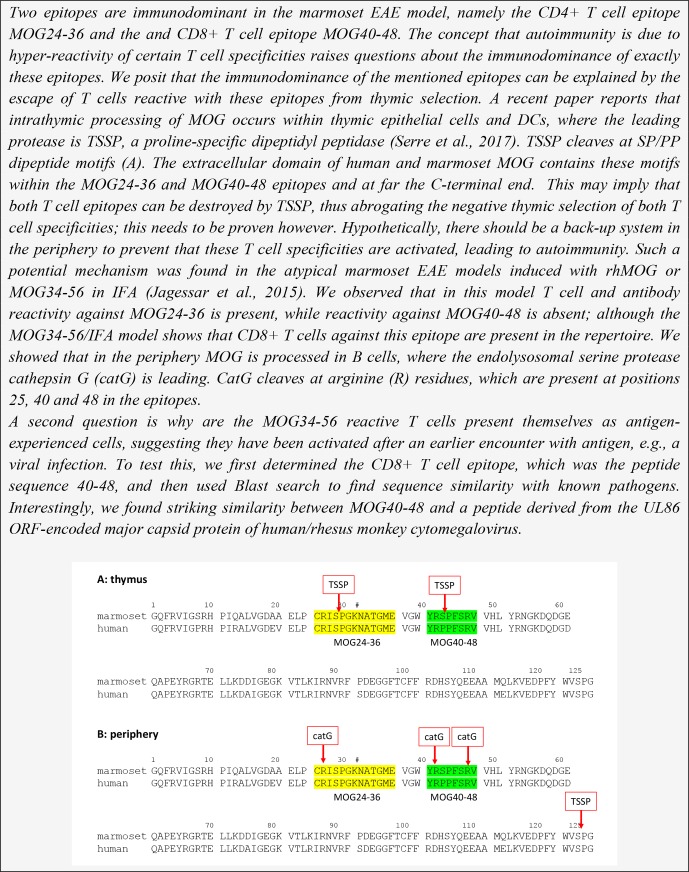
Why are the MOG24-36 and MOG40-48 immunodominant?

**Figure 8:**
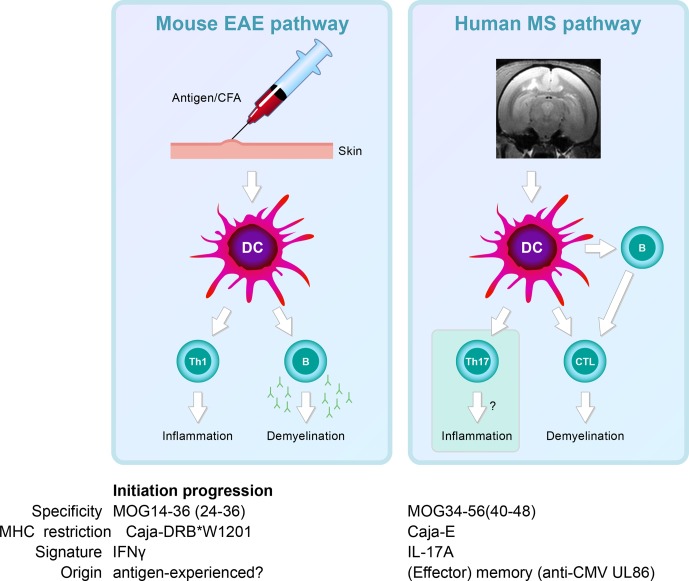
*Two autoimmune pathways.* EAE development in the rhMOG/CFA model involves two distinct pathogenic mechanisms. The 100 % EAE incidence (see Fig. 7a) maps to the MHC class II/Caja-DRB*W1201-restricted activation of Th1 cells specific for MOG epitope 24–36. The signature cytokine of this pathway, which essentially replicates mouse EAE models, is IFNγ. Early blockade of IL-12/IL-23 with a mAb against IL-12p40 abrogates the activation of this pathway. The observation that the EAE initiation pathway is activated in rhMOG/IFA model indicates that the responsive Th1 cells may be antigen-experienced cells. The variable EAE onset maps to another pathogenic mechanism, namely the MHC class I/Caja-E-restricted activation of CTL specific for MOG epitope 40–48, which shares almost complete sequence similarity with an epitope from the major capsid protein of CMV. The signature cytokine of this pathway is IL-17A. The characteristics and specificity of the CTLs suggest that they originate from the effector memory T cells that keep CMV under control. We hypothesize that the variable activation of the latter pathway is induced by antigens released from white matter lesions induced by the former pathway.

The immunological analysis of the rhMOG/CFA model revealed two distinct pathogenic mechanisms (Fig. 8). We found that the 100 % EAE incidence maps to a common autoimmune mechanism, namely the Caja-DRB*W1201-restricted activation of Th1 cells specific for epitope MOG24-36 (Brok et al., 2000). By contrast, we found that the variable EAE course was related to the degree of diversity of the T-cell response against MOG peptides (Kap et al., 2008). Counterintuitively, monkeys that progressed relatively fast to clinically definitive EAE were characterized by a broader T-cell reactivity with rhMOG peptides than slow progressor cases. This is opposite to mouse models, where EAE progression is associated with progressive diversification of autoreactive T-cell reactivity, a phenomenon known as “epitope spreading” (Vanderlugt et al., 1998). Our interpretation were that while fishing in the pool of MOG breakdown products, certain MHC alleles present in the repertoire of fast progressor monkey bind peptides that can activate T cells with the capacity to accelerate EAE development. Later studies showed a role of IL-7 in the pathogenic function of these accelerator T cells (Dunham et al., 2016). Candidate epitopes emerging from T-cell reactivity scans were MOG4-26, MOG34-56 and MOG64-86/74-96 (Brok et al., 2000; Kap et al., 2008). The encephalitogenic activity of MOG4-26 was not (yet) tested. For the other two, we observed potent encephalitogenic activity for MOG34-56, while MOG74-86/74-96 was inactive (Kap et al., 2008). Subsequent studies revealed that the core pathogenic epitope is MOG40-48 and that this epitope can be presented to (antigen experienced) cytotoxic T cells (CTLs) via Caja-E molecules (Jagessar et al., 2012d). Besides the MHC-E restriction of epitope recognition, these CTLs express the natural killer (NK) cell marker CD56 and the MOG40-48 epitope shares almost sequence similarity with a peptide derived from the major capsid protein of cytomegalovirus (Brok et al., 2007) we tentatively identified these CTLs as being potentially related to or similar to CMV-induced effector memory T cells (Pietra et al., 2003).

In summary, we showed two independent pathogenic mechanisms in the rhMOG/CFA marmoset EAE model: a mouse EAE-like pathway operating early in the disease and a novel pathway starting late in the disease and mediating EAE progression.

**Figure 9:**
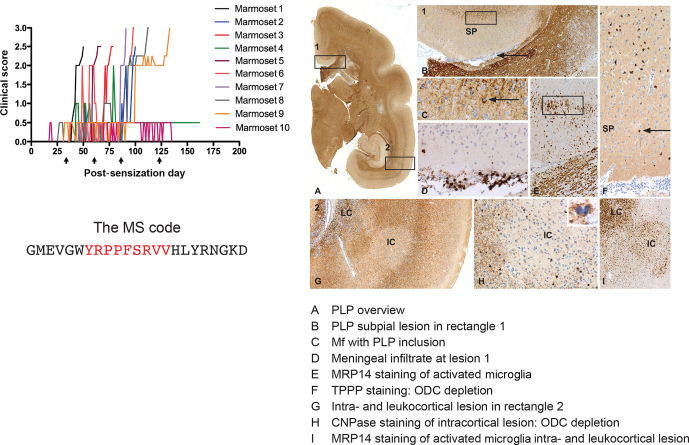
*Clinical and pathological aspects of an atypical EAE model induced with MOG34-56/IFA*. Immunization with MOG34-56/IFA at a 28 d interval (arrows) induces 100 % clinical EAE with a variable time of onset. Notice that the only information relayed to the marmoset's immune system is the sequence of 23 letters (CTL core epitope in red). Immunostaining for PLP shows lesions in the white and grey matter (A). Rectangle 1 indicates a demyelinated region in the cingulate cortex, which is characterized by complete demyelination (B), presence at the lesion edge of macrophages containing a PLP+ inclusion particle (C), activated MRP14+ microglia (E) and depletion of oligodendrocytes (F). Immune cells (CD3) were only detected in meninges. Rectangle 2 (A) indicates a leukocortical and an intracortical lesion (G), which are also depleted from oligodendrocytes (H) and contain abundant MRP14+ microglia. Mf is macrophage, TPPP is tubulin polymerization-promoting protein, ODC is oligodendrocyte.

Based on findings discussed above we immunized marmosets with MOG34-56 or MOG74-96, each formulated with CFA. Of these two, MOG34-56 emerged as the superior encephalitogenic peptide (Kap et al., 2008). Intriguingly, we observed 100 % EAE incidence in marmosets sensitized against MOG34-56 together with widespread demyelination in the white and grey matter of brain and spinal cord (Fig. 9), despite the absence of detectable levels of myelin-binding antibodies in the serum. In addition, we observed that CFA could even be replaced by IFA, which is only the mineral oil without mycobacteria (Jagessar et al., 2010). A comparable mechanism has not been found in any other EAE model, indicating that this might represent a novel pathogenic mechanism that may be confined to the pathogen-educated primate immune system. Indeed, characterization of T-cell responses in this model revealed that they phenotypically resemble IL-17A${}^{+ve}$ natural killer cytotoxic T lymphocyte (CD8${}^{+}$CD16${}^{-}$CD56${}^{+}$; NK-CTL) and that the core epitope (residues 40–48; henceforth indicated as MOG40-48) is presented via nonclassical Caja-E molecules. Furthermore, we observed cytotoxic activity of the NK-CTL towards B cells infected with EBV (strain 95-8) (Jagessar et al., 2012d). Intriguingly, NK-CTLs have been found in MS lesions in the vicinity of HLA-E expressing oligodendrocytes, suggesting cytotoxic interaction (Zaguia et al., 2013). The observation that the MOG40-48 sequence shares sequence homology with a peptide from the major capsid protein of cytomegalovirus (CMV) sheds light on the possible origin of the encephalitogenic NK-CTL, namely the repertoire of T cells that suppress exacerbation of CMV (Pietra et al., 2003; Mazzarino et al., 2005).

A serendipitous finding revealed which of the two pathways described in Box 3 can be associated with GM pathology. We observed in an atypical marmoset EAE model induced with recombinant human (rh) MOG formulated with incomplete Freund's adjuvant (IFA) the activation of T cells against MOG24-36 together with antimyelin antibodies. However, in these animals T- and B-cell reactivity with the MOG34-56 peptide was lacking (Jagessar et al., 2015). Histological analysis of the brain showed profound demyelination of white matter (Jagessar et al., 2015), while cortical grey matter demyelination was minimal (own unpublished results). This suggests a causal relation between the absence of autoimmunity against MOG34-56 and the absence of grey matter pathology. Indeed, sensitization of marmosets against the MOG34-56 peptide in IFA induced fulminant cortical grey matter demyelination, with a crucial role of EBV-infected B cells ('t Hart et al., 2017a, see below for further discussion).

The consideration that MS is obviously not elicited by injection of an antigen/adjuvant formulation but develops spontaneously raises at least two important questions:

1. What is the nature of the primary lesion that starts the release of myelin antigens?
2. What is the nature of the antigen-presenting cells (APCs) that capture and process these antigens, with a special focus on MOG, and present them to encephalitogenic T cell (response-to-injury paradigm)?

## 8 Considerations on the primary lesion

The review of immune responses against CNS myelin injury will be briefly interrupted here for a brief summary of our current insights into the primary lesion. The response-to-damage/inside-out paradigm is based on the assumption that some pathological event inside the CNS precedes the activation of autoreactive T and B cells that may be more responsive to the damage due to prior antigenic experience. In the EAE model the antigenic experience comes from active or passive immunization; in MS this might rather be infection with a pathogen sharing molecular mimicry with a CNS antigen.

Already in 1990, Terence Wilkin postulated in his primary lesion theory that “autoimmunity is a genetically predisposed immune hyper-reaction to the excess of autoantigen released from a primary lesion” (Wilkin, 1990). Shedding of fragments from aging myelin sheaths seems to be a normal physiological process (Safaiyan et al., 2016). Our own data show that this may not inevitably lead to autoimmunity, as binding to myeloid APCs is mediated by normally glycosylated MOG. It was observed that MOG isolated from a nonpathological brain binds to the C-type lectin receptor DC-SIGN (Garcia-Vallejo et al., 2014), which is expressed within the human CNS on microglia and within primate cervical lymph nodes (CLNs) on the phagocytes that have captured the myelin (Garcia-Vallejo et al., 2014). DC-SIGN is best known for its role in antigen capture and presentation by myeloid dendritic cells (mDCs). Ligand activation of DC-SIGN relays inhibitory signals to the dendritic cells (DCs), which keeps them in an immature, tolerogenic state (Geijtenbeek et al., 2004) and prevents the assembly of inflammasomes, which are needed for the production of bioactive IL-1β (Garcia-Vallejo et al., 2014). Note, studies in Alzheimer mice identified inflammasome activation in microglia as a crucial step towards neurodegeneration (Heneka et al., 2013).

The inflammasome response of cells is regulated by autophagy; more specifically, inflammasomes can be removed from cells via autophagy (Takahama et al., 2018). Mice defective in autophagy genes display intracellular accumulation of protein aggregates in the cytosol of neurons and hepatocytes. This raises the question of whether MS patients may be defective in autophagy. Although this subject has not been intensively studied, a publication by Igci et al. (2016) reported altered expression levels for several genes encoding autophagy-related proteins in MS versus healthy controls.

These (and other) findings led to the concept that myelin binding to phagocytes in draining lymph nodes is mediated via MOG, which via its N-linked glycan attached to Asn31 bind the C-type lectin receptor DC-SIGN. The response of the APC to myelin debris therefore depends on whether MOG exposed on the myelin particles is normally or abnormally glycosylated (see also 't Hart and van Kooyk, 2004). This concept may narrow the problem of autoimmunity initiation in MS to the question of what causes the disturbance of normal MOG glycosylation. Intriguingly, we observed that myelin produced under inflammatory conditions (exposure of oligodendrocytes to TNF-α) has a different glycan make-up and fails to counteract DC activation via Toll-like receptors (TLRs) (see Box 2).

**Box 4:**
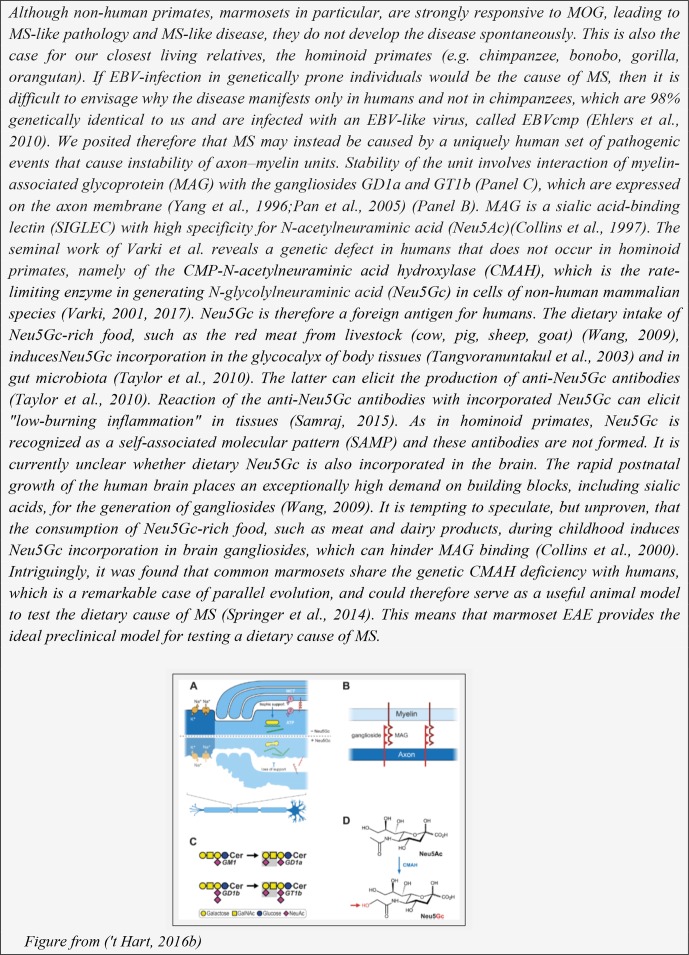
Why do humans develop MS and chimpanzees not? A hypothesis.

The first histological evidence of neuro-inflammation in MS is the presence of microglia aggregates in normal-appearing white matter in the absence of other inflammatory cells or demyelination. These aggregates are indicated as preactive lesions (van der Valk and Amor, 2009) a.k.a. microglia nodules (Singh et al., 2013) a.k.a. newly forming lesions (Barnett and Prineas, 2004), but likely represent the same pathological entity (Sato et al., 2015). Interestingly, similar aggregates of microglia were found in the EAE models in marmosets (Maggi et al., 2014) and in rhesus monkeys (Burm et al., 2016). In the marmoset EAE model the non-demyelinating inflammatory microglia nodules correlated with small-sized prelesional MRI-detectable blood–brain-barrier leakage and were “contaminated” with some immune cells (T cells, macrophages) (Maggi et al., 2014). It is currently unclear whether they are completely comparable to the pure microglia nodules in MS. The observation by Singh et al. (2013), that microglia nodules are formed around a degenerating axon, draws the interest towards the axo-myelinic synapse, a new functional concept for the axon–myelin unit (Micu et al., 2017). A graphic representation of the unit is depicted in Box 4. As discussed elsewhere, an intact unit is essential for adequate trophic support to high nutrient demanding axons ('t Hart, 2016b). We posit that these findings underlie the inside-out concept that the primary cause of MS may be instability of the axon–myelin unit (Stys et al., 2012). One can only speculate why this happens. It could have an internal cause, such as a genetic or metabolic defect and subtle molecular changes (Caprariello et al., 2018), or an external cause, which could be associated with a Western lifestyle ('t Hart, 2016b) (see Box 4), an infection or a combination of all features. It is pertinent to emphasize, however, that our choice for the inside-out paradigm as a working concept does not negate the possibility that MS is triggered by an external factor.

## 9 Antigen-presenting cells mediating the response-to-injury paradigm

### 9.1 Antigen capture by phagocytes in CNS draining cervical and lumbar lymph nodes

Conceptually, the cells that capture, process and present antigens to the immune system have a decisive influence on the way the immune system responds to injury. The original thought that the CNS lacks lymphatic vessels via which CSF and interstitial fluids can be drained to the lymphoid system needed to be adjusted after the discovery of such structures in the meninges that surround the brain and spinal cord (Louveau et al., 2015; Aspelund et al., 2015). Note, similar structures were found in the marmoset brain (Absinta et al., 2017). Via these lymph vessels, fluids draining from the brain and spinal cord respectively pass through the cervical and lumbar lymph nodes (CLNs and LLNs), where antigens might be captured by local APCs. Via in situ immunochemistry we could demonstrate the localization of myelin antigens within DC-SIGN+ phagocytic cells of EAE-affected mice and marmosets (de Vos et al., 2002). Moreover, we observed that surgical removal of CLNs and LLNs impairs the chronic relapsing EAE course in MOG-sensitized Biozzi/ABH mice (van Zwam et al., 2009). These findings suggest that the induction of T-cell reactivity against injury may take place in the CLNs and LLNs. Indeed, we have frequently detected ex vivo T-cell reactivity (proliferation) against MOG peptides in mononuclear cell suspensions from CLNs and LLNs of EAE marmosets.

### 9.2 B cells as requisite antigen-presenting cells (APCs)

For many years, translational therapy research in MS was mainly focused on the modulation or suppression of autoreactive CD4+ T cell functions (Ransohoff et al., 2015). However, in the past 10 years the field has changed rather dramatically, sparked by the discovery that depletion of B cells via administration of the anti-CD20 mAb rituximab had an unexpected positive clinical effect in RRMS (Hauser et al., 2008). This remarkable finding has since then been confirmed with two fully human anti-CD20 mAbs, ofatumumab and ocrelizumab (Barun and Bar-Or, 2012). The latter mAb even exerted a positive clinical effect in patients with primary progressive MS (PPMS) (Montalban et al., 2017). Collectively, these findings indicate that B cells have a much more prominent pathogenic role in MS than just the production of autoantibodies. We investigated via which mechanism(s) B cells exert their core pathogenic role in the marmoset EAE model and whether this role is executed by all mature B cells or only by a certain subset.

An alternative method for B cell depletion is by capturing cytokines that B cells need for survival and differentiation, i.e., BlyS/BAFF or APRIL (Dillon et al., 2006). To achieve this, the chimeric protein atacicept has been developed by joining TACI, the joint receptor for BlyS and APRIL on B cells, with an Fc fragment of human immunoglobulin G. Unexpectedly, atacicept showed no relevant clinical effect in RRMS and even worsened lesion activity, necessitating the termination of two clinical trials (Kappos et al., 2014).

These paradoxical clinical findings in MS sparked our interest in comparing the effect of mAbs against CD20, BlyS and APRIL in the marmoset EAE model; we used the models induced with rhMOG/CFA or with MOG34-56/IFA. As rituximab failed to bind marmoset B cells, we selected a clonal variant of ofatumumab (HuMab7D8), which cross-reacted with marmoset B cells. We observed that late-stage treatment (from psd 21, post-sensitization day) with the anti-CD20 mAb abrogated EAE development in marmosets immunized with rhMOG/CFA (Kap et al., 2010), whereas the anti-BlyS and anti-APRIL mAbs only moderately delayed EAE onset (Jagessar et al., 2012e). As depletion of B cells from the circulation was observed in both types of treatment, we focused our analysis on the secondary lymphoid organs (SLOs), i.e., lymph nodes and spleen. Intriguingly, we observed that in the monkeys treated with anti-CD20 mAb, but not in those treated with anti-BlyS or anti-APRIL mAbs, T cells within SLOs remained strongly positive for CCR7, indicating that their release into the circulation might have been impaired (Kap et al., 2014). Furthermore, we observed that in the monkeys treated with anti-CD20 mAb, but not in those treated with anti-BlyS or anti-APRIL mAb, the copy numbers of CalHV3 DNA, being a lymphocryptovirus of marmosets, was strongly reduced, indicating the depletion of CalHV3-infected B cells (Jagessar et al., 2013a).

Collectively, these data underlie the following concepts:

1. that B cells act as requisite antigen-presenting cells for the T cells that initiate and perpetuate EAE in marmosets,
2. that this role may be primarily executed by the CalHV3-infected B cell fraction.

This concept was further tested in the MOG34-56/IFA EAE model in which lesion development in WM and cGM occurs independent of anti-MOG antibodies (Jagessar et al., 2010). As expected, we also observed that the development of clinical signs and CNS pathology in this model was substantially reduced by the treatment with anti-CD20 mAb, hinting at a direct role of B cells in the activation of pathogenic T cells (Jagessar et al., 2012b).

### 9.3 Antigen processing and presentation

We then asked why LCV/CalHV3 infection of B cells is important for their role as APCs in the marmoset EAE model, in particular for presentation of the vulnerable MOG34-56 peptide to pathogenic T cells. Inspired by a seminal publication from Manoury et al. (2002), who first presented the concept of destructive processing of autoantigen (MBP) in thymic APCs as an explanation for why certain autoreactive T cells escape thymic selection, we proposed that this might also be the case for MOG ('t Hart et al., 2016). Our hypothesis was supported by recent studies in mice, indicating that autoreactive T cells specific for the MOG40-48 epitope escape negative thymic selection because the epitope is destroyed by a thymus-specific serine protease (TSSP), which is expressed in thymic APCs. TSSP cleaves at SP/PP residues within the MOG40-48 epitope (see Box 3). We proposed that peripheral activation of these escaped autoreactive T cells is prohibited by B cells expressing the lysosomal serine protease cathepsin G (catG). CatG cleaves the epitope at the Arg${}^{41}$ and Arg${}^{46}$ residues ('t Hart et al., 2016). We also proposed that this peripheral back-up tolerance mechanism might be impaired when the B cells are infected by EBV/CalHV3. This concept was tested in nonhuman primate B lymphoblastoid cell (BLC) lines, which were obtained by ex vivo infection of blood mononuclear cells with EBV isolate 95-8 (for marmoset) or herpesvirus papio (for rhesus monkey).

We observed that infection of B cells with the EBV-related lymphocryptoviruses (LCVs) induced a variety of cell biological changes that potentiate their pathogenic role in the EAE model. These include the following:

- *Malignant transformation.* Transformed B cells are constitutively in an activated state and are relatively resistant to apoptosis. Moreover, they have the capacity to migrate across the BBB into the CNS (Pender, 2003). We indeed observed that infusion of marmosets with autologous BLC, which were prepulsed in vitro with MOG34-56, evoked mononuclear cell infiltrates in meninges and small-sized perivascular inflammatory lesions (Haanstra et al., 2013c; Jagessar et al., 2013a).
- *Immune evasion.* One of the immune escape mechanisms employed by LCVs is to avoid T-cell recognition of viral antigens by cytotoxic T cells through mitigation of antigen presentation via MHC class Ia (MHC-A, -B, -C) and MHC class II (MHC-DR) molecules (Ressing et al., 2008). The absence of MHC class I molecules evokes an attack by NK cells on the B cells, which can be repelled by the upregulation of MHC-E molecules. To achieve this, MHC-E must be occupied by epitopes expressing a so-called QdM motif, which directs the interaction of MHC-E towards inhibitory CD94-NKG2A heterodimeric NK receptors (Vales-Gomez et al., 1999). Binding of peptides lacking a QdM binding motif directs MHC-E binding to activating CD94/NKG2C heterodimeric NK receptors (Miller et al., 2003). Interestingly, the MOG40-48 epitope was found to bind with high affinity to HLA-E and Caja-E molecules expressed by transfectant K562 cells (Jagessar et al., 2012d).
- *Antigen cross-presentation.* LCV-infected B cells have the capacity to cross-present peptide antigens via MHC class Ib/Caja-E molecules to CD8+ cytotoxic T cells (Jagessar et al., 2012d).
- *Citrullination.* The upregulation of peptidylarginine deïminase (PAD) enzymes in LCV-infected B cells enables the conversion of arginine residues into citrullines (Ireland and Unanue, 2011, 2012).
- *Autophagy.* The autophagy pathway seems to be constitutively activated in LCV-infected B cells. Evidence from EBV-infected B cells suggests that constitutive activation is induced by the viral antigen LMP2A, which drives B cell function and survival (Caldwell et al., 1998). LMP2A replaces the B-cell receptor as inducer of a signaling cascade also involving Bruton's tyrosine kinase (Btk) (Merchant and Longnecker, 2001).

Each of the above-listed capacities of LCV-infected B cells can contribute to the still elusive association of MS with EBV infection, especially around adolescent age. However, evidence was found that the combination of antigen citrullination and autophagy activation underlies the presentation of the potent encephalitogenic MOG34-56 peptide by EBV-infected B cells (Ireland and Unanue, 2011). Our specific interest for antigen processing and presentation in B cells was sparked by some remarkable observations. First, we observed that immunization with MOG34-56/CFA elicited severe clinical EAE in rhesus monkeys (Brok et al., 2007), while in monkeys immunized with MOG34-56/IFA neither clinical signs nor T- or B-cell responses against the peptide could be measured (own unpublished observation). However, when the peptide was administered via ex vivo-pulsed LCV-infected B lymphoblastoid cell lines, T-cell reactivity with the peptide could be measured ex vivo. Second, we observed that T- or B-cell reactivity with the MOG34-56 peptide was conspicuously absent in marmosets immunized with rhMOG/IFA (Jagessar et al., 2015), while T- and B-cell immunity is clearly detectable in marmosets immunized with MOG34-56 peptide in IFA (Jagessar et al., 2010). Moreover, we observed that infusion with LCV-infected B lymphoblastoid cells pulsed ex vivo with the peptide elicited T- and B-cell reactivity in marmosets (Jagessar et al., 2013a).

**Box 5:**
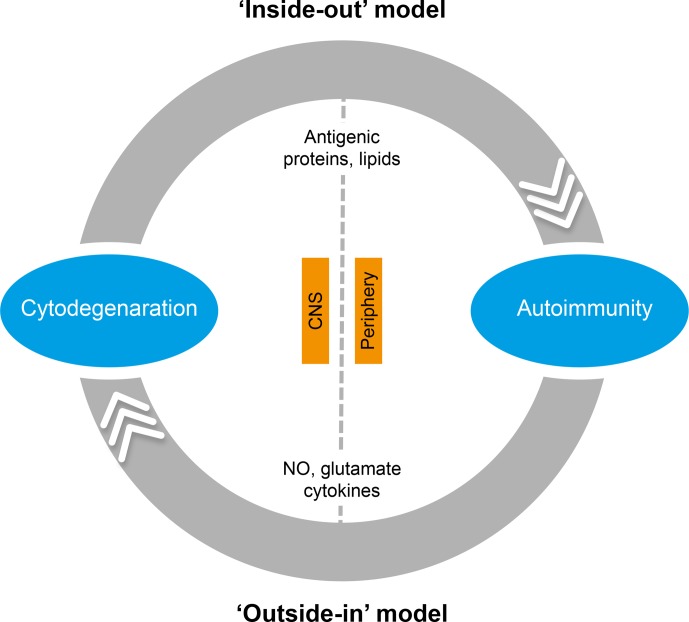
Destructive and productive processing of MOG34-56.

Collectively, these findings led to us to hypothesize that the MOG40-48 epitope might be destroyed during normal processing of rhMOG in APCs and that LCV infection of B cells might cause the conversion of destructive processing of the MOG40-48 epitope into productive processing of the epitope from the immunizing MOG34-56 peptide and presentation via MHC-E molecules (see Box 5 for details). This concept was tested in EBV-infected and CpG-stimulated human B cells (Morandi et al., 2017a). An important discovery was that LCV infection of B cells induces activation of the autophagy pathway (see Box 6). In brief, we observed that the replacement of only the Arg${}^{46}$ residue in the MOG40-48 epitope by citrulline had a profound effect on the degradation of the peptide by catG in intact EBV-infected B cells, while the Arg${}^{41}\to$ citrulline substitution exerted only a minor effect. In particular, we observed that activation of the autophagy pathway with the mammalian target of rapamycine (mTOR) inhibitor reduced degradation of the peptide, while inhibition of autophagy with 3-methyladenine (3-MA) increased degradation. Finally, we could detect localization of rhMOG within autophagosomes, which were visualized by staining cells for the docking autophagosome molecule microtubule-associated protein light chain 3 (LC3) (Morandi et al., 2017b).

**Box 6:**
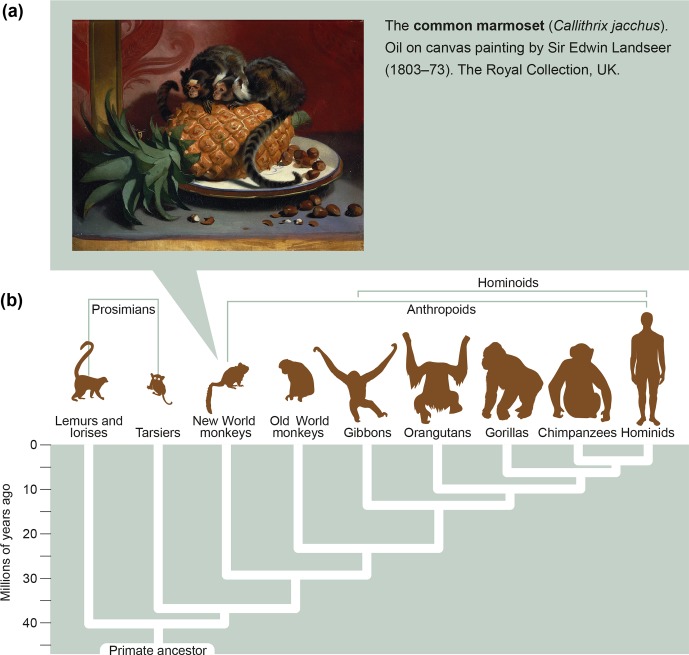
Autophagy.

Our interpretation of these data was that citrullination of Arg${}^{46}$ may protect a putative F-LIR (LC3-interacting region) motif within the MOG40-48 epitope (xSxFSRVx) against cleavage by catG (see Birgisdottir et al., 2013). Conceptually, the association of the MOG34-56 peptide with autophagosomes may explain the protection of the MOG40-48 epitope against fast degradation in lysosomes, which leads to enhanced immunogenicity (Delamarre et al., 2006) and facilitates cross-presentation (Munz, 2009). Moreover, evidence suggests that HLA-E localizes in autophagosomes, where uploading with the peptide could take place (Camilli et al., 2016). Collectively, these data provide a plausible novel explanation for the EBV-MS association and for the pathogenic role of the EBV-infected B cell.

In discussions about the causal association of EBV infection with MS the counter argument is often raised that the EBV infection prevalence in the human population (90 %) is much higher than the prevalence of MS (±0.1 %). Data from the marmoset EAE model warrant the hypothesis that the MS risk may not map to events triggered by the infection, but to capacities that B cells with a meaningful specificity for MS acquire by the infection with EBV. In this context it is important to notice that in EBV carriers, the frequency of B cells that contain the virus was estimated at between 1 and 50 per 10${}^{6}$, i.e., < 0.005 % (Khan et al., 1996). So the question of whether myelin-reactive B cells are more often infected with EBV in MS than in healthy controls or patients with other diseases arises. We are not aware of any published direct evidence. However, it has been reported that autoantibody responses against native MOG were found in about 20 % of adolescents with infectious mononucleosis, i.e., symptomatic EBV infection, while this antibody specificity was not found in healthy control cases (Kakalacheva et al., 2016). MOG-specific IgG responses declined after clinical resolution of infectious mononucleosis (IM), indicating the antibodies were either produced by EBV-infected B cells themselves or a bystander product of noninfected B cells induced by factors from EBV-infected B cells. Note, MOG-specific IgG antibodies are found in 40 % of adolescents with an autoimmune CNS demyelinating disease (ADEM, MS, NMOSD), but not in healthy control subjects. These data hint at the possibility that MOG-specific B cells are more frequently infected with EBV in MS than in healthy age-matched adolescent controls.

**Figure 10:**
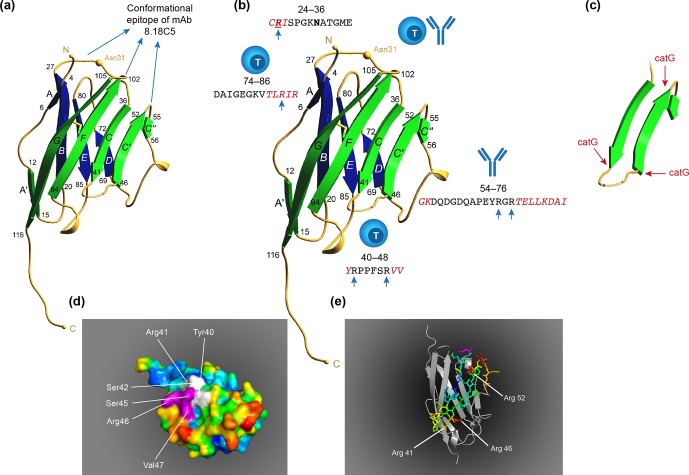
*T and B cell epitopes plotted on the 3-D structure of MOG monomer*. **(a)** The conformational antibody epitope is formed by the three loops that connect the B–C, C'–C” and F–G β sheets. Notice that the B–C connecting loop (residues 27–36) overlaps with the CD4 T cell epitope and contains the Asn31 residue to which in the native molecule the N-linked glycan is attached. **(b)** The positions of the three dominant T cell epitopes and a linear antibody epitope are indicated. Notice that these epitopes all overlap with β-sheet connecting loops. **(c)** An excision of the critical MOG34-56 peptide, which appears to be composed of two large antiparallel β sheets and a small one. Notice that the three critical Arg residues in the MOG34-56 peptide where the peptide can be cleaved by cathepsin G (positions 41, 46 and 52) are located at contact points of loop and β sheet. **(d)** Depicts a space-filling model of monomeric MOG *(pdb accession number 1PKO)* in molecular surface representation, colored according to B factor (blue low rms/rigid; red high rms/flexible. The surface-exposed MOG40-48 epitope (YRSPFSRVV) is indicated in white/purple. The P43 and F44 residues, which stick out of the plane towards the reader, are not resolved in the structure, probably due to the high flexibility of this part of the sequence resulting in a diffuse diffraction pattern (Breithaupt et al., 2003), the V48 residue is buried in the interior of the protein, and therefore not visible. The putative LIR motif (xSxF${}_{43}$SRV${}_{47}$), which is part of the 40–48 epitope is shown in purple. The surface exposure of this motif enables interaction with the LC3 docking molecule of autophagosomes. **(e)** In a ribbon representation of monomeric MOG the three Arg residues are highlighted. It is clear from this figure that the Arg${}^{46}$ and Arg${}^{52}$ residues stick out while the Arg${}^{41}$ residue is somewhat buried.

### 9.4 Transfer of antigen from myeloid APCs to B cells?

**Figure 11:**
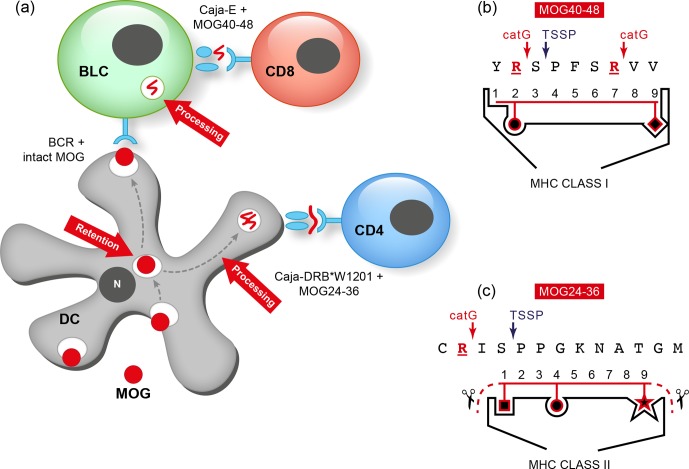
*Concept for T cell activation within the CLNs*. **(a)** MOG draining via interstitial fluid (ISF) or cerebrospinal fluid (CSF) to the CLNs is captured by DC-like phagocytic cells (de Vos et al., 2002). **(b, c)** T cells against the two dominant epitopes (24–36 and 40–48) are probably not deleted during thymic negative selection as both epitopes are vulnerable to destruction by TSSP, which cleaves at SP/PP residues present in both epitopes (Serre et al., 2017). Processing of MOG in myeloid APCs and B cells is led by catG, which cleaves at arginine (R) residues (Jagessar et al., 2016). This mechanism prevents peripheral activation of autoaggressive T cells that have escaped thymic selection. **(b)** The peptide sequence left after cleavage of the MOG40-48 epitope at Arg${}^{41}$ and Arg${}^{46}$ (the 4-mer 42–45) is too short for filling the peptide-binding cleft of the MHC class I molecule. In the BLC, however, the MOG40-48 epitope is protected against destructive processing. **(c)** Cleavage of the MOG24-36 epitope at Arg${}^{25}$ may have little effect as a peptide of sufficient length (the 9-mer 26–34) is left intact for filling the peptide-binding cleft of the MHC class II molecule.

The observations discussed thus far imply that myelin antigens (MOG for example) are captured by myeloid APCs in CLNs or LLNs, while the B cell depletion studies reveal a key function of LCV-infected B cells in the EAE model. This somewhat paradoxical situation can be understood when MOG is in some way transferred from the myeloid APCs to the LCV-infected B cells for further processing. However, in the absence of direct evidence, this explanation can only be speculative. The fact that pathogenic anti-MOG antibodies bind to a conformational epitope formed by three loops at the apical tip of the molecule (Fig. 10a), implies that the B cells should receive via their BCR conformationally intact MOG from the DCs. A publication by MacPherson (MacPherson et al., 1999) seems to be important in this context. The author reports that part of the protein antigens that are taken up by DCs is not processed but transferred to B cells in the intact form (Fig. 11). In the rhMOG/IFA model only CD4+ T cell activation against the MOG24-36 epitope could be detected, while the MOG34-56 peptide seemed to be destroyed, as neither T cell nor antibody reactivity against this peptide could be detected (Jagessar et al., 2015). The model in Fig. 11 may explain how the two EAE pathways can be activated independent from each other. When MOG is processed through endolysosomal catG within the DC-like phagocytes, a sufficient part of the MOG24-36 epitope remains intact for occupation of the Caja-DRB*1201 epitope binding cleft, while the MOG40-48 epitope is destroyed. Part of the MOG molecules is transferred relatively intact (possibly because the Arg residues are citrullinated) to the B cells, which via the infection with LCV have acquired the capacity to cross-present the protease-sensitive MOG40-48 epitope via the autophagy pathway (Box 6) and Caja-E to CD8+ T cells (reviewed in 't Hart et al., 2016). It is tempting to speculate that Arg residues in critical epitopes of MOG released from lesions are citrullinated before they are captured by the APC, thus ensuring that epitopes resist destructive processing. Molecular modeling shows that the Arg${}^{46}$ and Arg${}^{52}$ residues are surface-exposed on MOG, while the Arg${}^{41}$ residue is buried (Fig. 10d). The accessible Arg${}^{46}$ and Arg${}^{52}$ residues can be citrullinated enzymatically via PAD or iNOS.

Regarding PAD, it was discussed that an increasing proportion of the myelin antigens in the MS brain is citrullinated through the upregulation of PAD2 by inflammatory factors and an increase in [${\mathrm{Ca}}^{2+}$] as essential cofactor (Carrillo-Vico et al., 2010; Bradford et al., 2014). Regarding iNOS, it was shown by Dunham et al. (2017a) that enzymes involved in oxyradical (${\mathrm{NOX}}_{2}$) and nitric oxide (iNOS) production are upregulated in the inflammatory active lesions in marmoset EAE brain. During ${\mathrm{NO}}^{*}$ synthesis, arginine is converted into citrulline, but it needs to be tested whether this can also occur when Arg is embedded in a peptide or protein (Rath et al., 2014).

In the mouse EAE model, the Arg residues are contact sites for the T cell antigen receptor. Hence, their post-translational modification (PTM) potentially reduces the amount of available antigen for the induction of relapses by pro-inflammatory T cells. This notion underlies the new concept that as a consequence of PTM the autoreactive T- and B-cell-driven inflammatory demyelination will gradually fade out and be replaced by a pathogenic process inducing progressive disease.

## 10 A non-immunological role of B cells in (progressive) MS

### 10.1 B cells in progressive MS

Data discussed thus far posit a central pathogenic role of LCV-infected B cells in the autoimmune pathway(s) leading to cortical grey matter (cGM) pathology (reviewed in 't Hart et al., 2017a). Their originally proposed role is the activation of effector memory CTL specific for MOG40-48, which can kill oligodendrocytes ('t Hart et al., 2017a). However, in contrast to observations in MS lesions (Zaguia et al., 2013), co-localization of T cells with MHC-E expressing oligodendrocytes could not yet be detected in the marmoset model. In fact, T cells were rare, if present at all, in subpial cGM lesions, but they were frequently found in the adjacent meninges ('t Hart et al., 2017a).

We were inspired by the concept that the interaction of T and B cells in meningeal infiltrates might produce (a) cytopathic factor(s) capable of directly inducing cGM demyelination (Howell et al., 2011; Choi et al., 2012; Gardner et al., 2013). In the rat model this activity was found to be mediated by pro-inflammatory cytokines, IFNγ and TNFα in particular (Gardner et al., 2013). Intriguingly, data from Lisak et al. (2012) suggest yet another mechanism, namely that oligodendrocyte death may be induced by B-cell-derived large molecular factors (Lisak et al., 2012), probably even extracellular vesicles (poster at American Academy of Neurology 2017 meeting; see https://www.youtube.com/watch?v=-3k1ubpq0Zo, last access: 17 April 2019). This theory is in remarkable agreement with observations reported 40 years ago on the detection of a high molecular transmissible factor in the MS brain, termed MS-associated agent (MAA), that is absent in the healthy brain and that was found to induce depression of neutrophils in mice (Koldovsky et al., 1975; Editorial, 1976).

These findings prompted the following experiments:

1. to test whether infusion of autologous LCV-infected B cells prepulsed in vitro with MOG34-56 peptide induces EAE pathology in rhesus monkeys and marmosets
2. to examine whether the interaction of LCV-infected B cells with or without MOG34-56 peptide with T cells from EAE marmosets in vitro elicits production of cytotoxic factors that may be implicated in cGM demyelination (Gardner et al., 2013).

Concerning study 1, we observed the formation of T and B cells containing meningeal infiltrates (in both species), together with small-sized perivascular inflammatory lesions (Jagessar et al., 2013a; Haanstra et al., 2013c).

Concerning study 2, we observed peptide-dependent production of IL-17A, while IFNγ and TNFα were produced at a negligible level (Dunham et al., 2017c), which essentially reproduces earlier findings in the EAE model induced with MOG34-56/IFA (Jagessar et al., 2012d). The combination of these observations indicates that MOG34-56 loaded LCV-infected B cells are indeed capable of eliciting neuro-inflammation, but that cytokines implicated in cGM demyelination in the rat model seem not to be produced in sufficient quantity for eliciting oligodendrocyte death in primate EAE.

**Figure 12:**
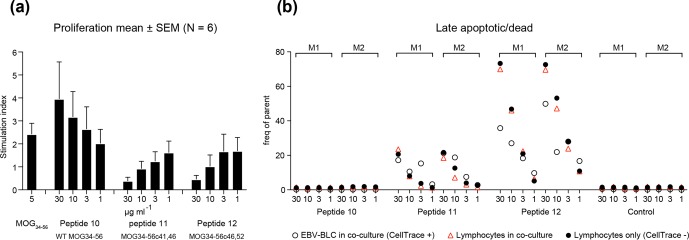
*Antigenicity and cytotoxicity of citrullination MOG34-56.* EBV-BLC cocultures with lymph node cells from EAE marmosets. **(a)** Marmoset EBV-induced BLCs were lethally irradiated and incubated for 1 h with titrating concentrations of unmodified MOG34-56 (peptide 10), or peptide citrullinated at positions 41+46 (peptide 11) or positions 46+52 (peptide 12). Subsequently, lymph node or spleen cells from marmosets immunized with MOG${}_{\text{34–56}}$ were added. T-cell responses to the peptides were assayed by proliferation and are expressed as stimulation index per culture condition. The experiment was conducted with N=6 (marmosets) and with three biological replicates. Data are presented as mean ± standard error of the mean (SEM). **(b)** To test which cell type is targeted by the peptides, EBV BLCs (from two marmosets, M1 and M2, respectively) were incubated with Celltrace dye before incubation with peptide (white circles) and mixture with the spleen/lymph node cells (red triangles). Lymphocytes that are not subjected to coculturing were used as controls (black circles). Cultured cells were harvested and stained for Annexin V as a marker of late apoptotic/dead cells. Final analysis was done utilizing flow cytometry.

There is another argument against the induction of oligodendrocyte depletion by T-cell-derived cytokines. The necessity to citrullinate the Arg residues within the MOG40-48 epitope for protection of the immunizing MOG34-56 peptide against destructive processing may affect T-cell recognition of the epitope. As was already mentioned, it has been shown in B6 mice that the Arg residues at positions 41 and 46 contact the antigen receptor of I-A${}^{b}$ restricted encephalitic CD4+ T cells and that T cell lines raised against the noncitrullinated epitope cannot be stimulated by the citrullinated epitope vice versa (Carrillo-Vico et al., 2010). We tested whether the same residues are needed for T-cell recognition in the marmoset model. Indeed, we found that while the unmodified peptides induced dose-dependent proliferation of T cells from marmosets immunized with MOG34-56/IFA, peptides with the Arg residues at positions 41+46 or 46+52 replaced by citrulline seem to exert cytotoxic activity (Fig. 12). It is therefore posited that the incrementing citrullination of myelin inevitably leads to reduced autoimmune activation and an increasing role of a novel B-cell-mediated cytopathic mechanism, which is discussed in the next paragraph.

### 10.2 A novel EBV B-cell-dependent cytopathic mechanism

The observations discussed thus far seem to dispute the concept that the depletion of oligodendrocytes in cGM lesions occurs by physical interaction with cytotoxic T cells or by T-cell-derived cytotoxic factors. Hence, we tested an alternative option, namely whether the MOG34-56 peptide itself or a metabolite thereof might be the elusive cytopathic factor.

Figure 10a depicts the 3-D configuration of monomeric human MOG as elucidated by Breithaupt et al. (2003). The structure is rich in β sheets, which are connected by α-helical loops. Indicated is the conformational epitope to which the monoclonal antibody 8.18C5 binds, with which demyelination has been induced in marmosets with inflammatory EAE (Genain et al., 1995). In Fig. 10b the position of the linear T cell and antibody epitopes are indicated. Note that the two dominant T cell epitopes (MOG24-36 and MOG40-48) overlap with β-sheet connecting loops. The encephalitogenic sequence 34–56 comprises three β sheets (C, C', C”), which are connected by α-helical loops (41–46 and 52–56) (Fig. 10c). Notice also that the Arg${}^{41}$, Arg${}^{46}$ or Arg${}^{52}$ residues are positioned where the loops and sheets are connected. Hypothetically, cleavage by catG at these positions separates the β sheets.

We observed the following: (1) replacement of these residues by citrulline protects the MOG34-56 peptide against cleavage by catG and (2) that citrullination promotes spontaneous aggregation of the peptide in the following order: MOG34-56 < MOG34-56c41,46 ≪ MOG34-56c46,52. Moreover, the oligomeric aggregates show amyloid-like seeding behavior (Araman et al., 2019).

**Figure 13:**
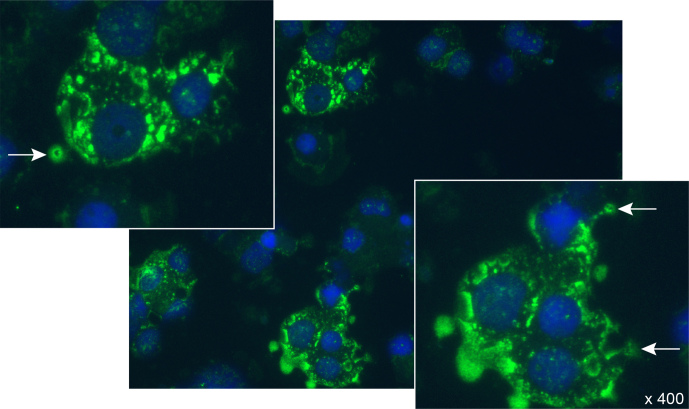
*LC3 staining of EBV-infected human B lymphoblastoid cells (BLCs).* Cytospin preparations of stably growing cells were prepared, fixed and stained with anti-LC3 + GAM-FITC conjugate. Arrows point to small LC3+ bodies, which seem to be shed from the BLCs.

These findings may shed an entirely new light on the role of LCV-infected B cells in cGM demyelination. It has been well-established that EBV-infected B cells secrete antigen-containing vesicles for spreading of an immune response (Raposo et al., 1996). The notion that the MOG34-56 peptide associates with autophagosomes via LC3 raises the question of whether LCV-infected B cells might secrete LC3+ vesicles. Figure 13 shows a picture suggesting that this may indeed be the case.

The next question was whether the vesicles contain MOG34-56 peptide. This was tested using synthetic peptides containing a bio-orthogonal group for attachment of a fluorochrome to peptide inside (fixed and permeabilized) cells. The peptides were unmodified (CA-1) or contained citrulline at the positions 41 and 46 (CA-2) or 46 and 52 (CA-3). Titrating doses of the peptides were fed to human EBV-infected BLCs for 48 h in serum-free medium.

**Figure 14:**
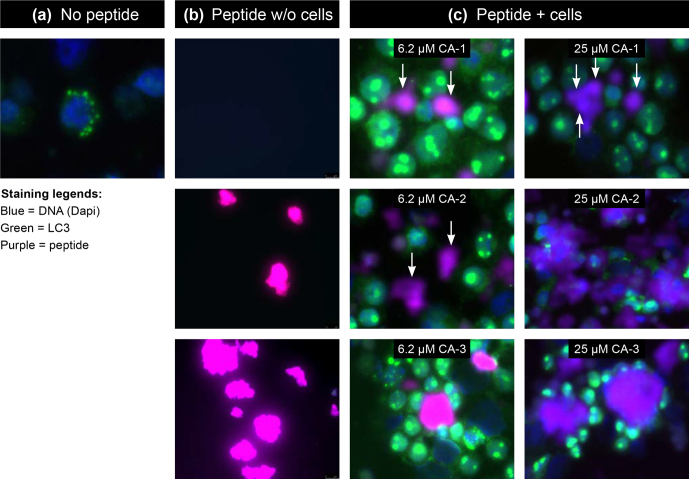
*Monitoring the uptake and aggregation of bio-orthogonal, site-specific citrullinated MOG peptides via confocal microscopy*. Human EBV-infected BLCs were incubated for 48 h with either none (peptide conc. 0 µM), 6.2 or 25 µM of unmodified MOG34-56 (peptide 10), or peptide citrullinated at positions 41+46 (peptide 11) or positions 46+52 (peptide 12) (highlighted in yellow). Cells were fixed with 4 % paraformaldehyde and processed for immunofluorescence with the following primary antibodies; the nucleus was stained with DAPI (blue) and LC-3 was used as an autophagosome marker (green). The bio-orthogonal peptides were stained using CuAAC chemistry with azide Alexa-647 (Thermo Fisher, the Netherlands).

Figure 14 shows stainings of cytospin preparations from BLCs incubated with 6.2 or 25 µM peptide in serum-free medium. Several noticeable observations can be made:

1. Column (a) shows that BLC incubated without peptide express only low staining of LC3.
2. Column (b) shows the same incubation conditions of the peptides, but without BLC. In the absence of BLC no precipitate of CA-1 is detectable, while aggregates of peptides CA-2 and CA-3 were spun down on the glass slides.
3. Column (c) shows BLC incubated with two doses of CA-1, -2 and -3, specifically 6.2 and 25 µM. Incubates of CA-1 with BLC contained globular structures staining positively for the peptide (arrows) are detectable between high-LC3 expressing cells. These structures do not contain a detectable nucleus (DAPI staining), while preliminary data show that peptide is enclosed within a membrane that binds anti-MHC class II (not shown). This may imply that the spherical structures are vesicles that have budded off from the B cells. The reason why the vesicles were spun down at relatively low centrifugal speed (3 min at 28 g) may be that they are attached to the much heavier nucleated BLC. At the higher peptide concentration LC3 expression of BLC was reduced and globular structures associated with diffuse aggregates of CA-1 were detectable.
  Also in the incubations with peptide CA-2 (citrullination at positions 41 and 46), albeit at lower concentration than CA-1, globular structures associated with diffuse aggregates of CA-1 were detectable with only scarce LC3+ BLC.
  In the incubations of CA-3 (citrullination at positions 46 and 52) with the B cells mainly large-sized aggregates were found surrounded by LC3-expressing BLCs.

These data strongly suggest that the CA-1 peptide has successfully survived passage through the B cells, which according to previous insights (Jagessar et al., 2016; Morandi et al., 2017a) implies that position 46 has been citrullinated, possibly via secreted PAD. In the cultures containing the globular structures, reduction of LC3 staining was observed, which points at a possible toxic effect.

**Figure 15:**
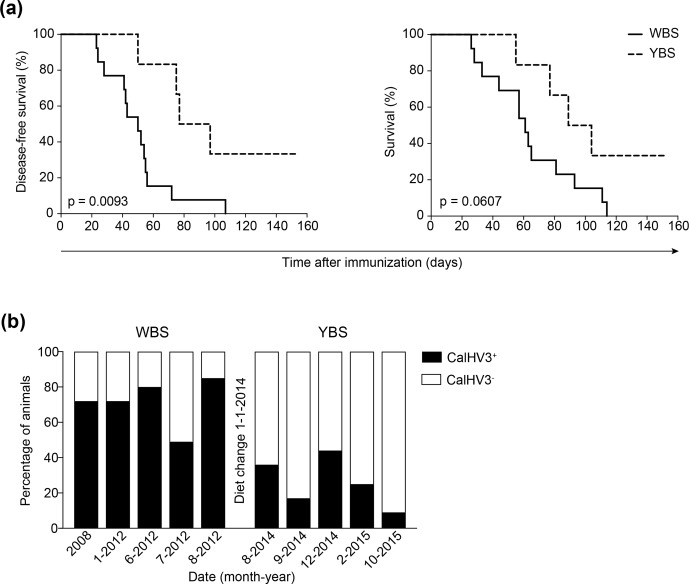
*Effect of the dietary modification (2014) on incidence of EAE and CalHV3 infection in the BPRC marmoset colony.* Since the discovery in 2012 that full-blown clinical EAE could be induced with the rhMOG/IFA model, 19 marmosets have been tested. **(a)** Time to clinical score 2 (disease-free survival) or the clinical endpoint (overall survival) of monkeys tested before (n=13 marmosets, water-based supplement is WBS) and after (n=6 marmosets) the introduction of the yogurt-based supplement (YBS). The graphs show a significant effect of the dietary modification on the incidence and course of EAE (log-rank test). **(b)** Periodic blood samples were collected from the colony at the dates on the x axis and tested for the presence of CalHV3 DNA using qPCR. The percentages of monkeys tested positive and negative for the viral DNA are given on the y axis. The figure shows that after the dietary change the percentage of monkeys in which CalHV3 could be detected was reduced from ±70 % to ±30 %.

In summary, we make the following four points:

1. We observed that immunization of marmosets with MOG34-56/IFA activates a pathway that leads to demyelination of white and cortical grey matter of the brain. The heart of this nonclassical pathogenic mechanism is formed by CD8+CD56+ effector memory cytotoxic T cells, which are activated by LCV-infected B cells presenting the epitope MOG40-48 via Caja-E molecules (Jagessar et al., 2010, 2012d). The similarity with data from the McGill group motivated us to posit that the CTL induce demyelination by killing oligodendrocytes (Zaguia et al., 2013). This concept was challenged by the finding that the MOG40-48 epitope is highly sensitive to destructive processing by cathepsin G, a leading endolysosomal protease in autoantigen processing in B cells (Burster et al., 2004).
2. We observed a special relevance of the citrullination of the Arg46 residue, which is located within a surface-exposed putative F-LIR motif that enables association of the immunizing MOG34-56 peptide with autophagosomes, as shown in EBV-infected human B lymphoblastoid cells fed with the peptide. In this way the peptide is protected against fast endolysosomal degradation, which enhances immunogenicity (Delamarre et al., 2006). The observations that treatment of the cells with rapamycine, a stimulator of autophagy, enhances protection of the peptide against degradation and treatment with 3MA, an inhibitor of autophagy, enhances degradation of the peptide support this concept (Jagessar et al., 2016; Morandi et al., 2017a).
  The replacement of arginine residues by citrullines impaired T-cell stimulation (proliferation). This finding argues against demyelination induction via cytotoxic T cells.
3. The paradoxical situation that citrullination of the Arg${}^{46}$ residue is essential for epitope survival in B cells but impairs TCR recognition necessitated the formulation of a new concept. Figure 15 shows staining of human EBV BLCs with an anti-LC3 mAb. Besides the expected punctate staining pattern, representing autophagosomes, LC3+ vesicles were also seen, which seem to have been shed from the cells. It is possible that these are extracellular vesicles (EVs) that BLCs produce for spreading ingested antigen with the aim to enhance the immune response (Raposo et al., 1996). Conceptually, citrullinated MOG34-56 can gain access to these LC3+ EVs via the association with the autophagy pathway, where they may form aggregates (to be determined).
4. Another line of research involved the serendipitous discovery that citrullinated MOG34-56 spontaneously aggregates to multimeric structures, which display seeding behavior comparable to amyloid-β. Note, the peptide contains three Arg residues, which are at positions 41, 46 and 52. As discussed above, the Arg${}^{46}$ residue mediates interaction of the peptide with LC3. Substitution of the Arg${}^{41}$ or Arg${}^{52}$ residue induces self-aggregation, although MOG34-56c46,52 is more aggregation-prone than MOG34-56c41,46.

Based on these collective data I posit that EBV-infected B cells may function as a Trojan horse in the progressive phase of MS, bringing MOG peptide oligomeric aggregates into the CNS and releasing them locally as packages within cytotoxic vesicles.

## 11 Studies in the effector arm of the marmoset EAE model

It was already recognized in the beginning of the model development that the human-like architecture of the marmoset brain and the MS-like histology of the EAE lesions might be relevant for studies on the monitoring of brain pathology development and treatment with magnetic resonance imaging (MRI) ('t Hart et al., 1998, 2004, 2006).

Our group at BPRC has developed a variety of sequences for visualizing and quantifying MRI sequences that were subsequently used for testing the radiological and clinical effects of therapeutic antibodies against human IL-12p40 and CD40 ('t Hart et al., 2005b, c). In addition, we used magnetic resonance (MR) spectroscopy of 24 h urine samples collected at 7 d intervals during the disease course. The demonstration that urine samples collected before, shortly after and late after EAE onset have different “metabolite fingerprints” indicates the presence of urinary biomarkers for the different pathological processes ('t Hart et al., 2003). However, the primary focus of our work has been on the afferent arm of the disease, being the initiation and perpetuation of the autoimmune mechanisms that cause the pathology. We mainly used the information from the therapy trials to assess the relevance of an immunological mechanism for MS and to adjust the model where needed ('t Hart et al., 2014, 2017b).

The effector arm of the disease has been studied in great detail by others. In studies by Absinta et al. (2016) and Maggi et al. (2017) a myelin-induced marmoset EAE model was examined with high-contrast MR images recorded at 7 T to monitor the initiation and development of lesions in the white matter. An extensive discussion of results obtained by these authors is beyond the scope of this monography. Hence, they will be briefly summarized. For more detail the interested reader is referred to publications from this group (see reviews by Absinta et al., 2016; Maggi et al., 2017, and references to primary publications therein).

As discussed above, it is highly difficult to assess whether inflammation precedes or follows lesion onset in the MS brain. In brief, analysis of the temporal development of brain white matter lesions in a myelin-induced marmoset EAE model show that lesions start as a small-sized focal enhancement of blood–brain barrier permeability developing into an acute inflammatory lesion (< 1 week old), which is cuffed around a central parenchymal vein and is composed of Iba-1+ microglia, CD3+ T cells, MRP14+ macrophages and (few) CD20+ B cells (Maggi et al., 2014). In lesions of older age (1 to 5 weeks), indicated as subacute, permeability of the blood–brain barrier was substantially reduced and demyelination was clearly noticed. The lesions typically contained Iba-1+ microglia, MRP14+ macrophages, CD3+ T lymphocytes and GFAP+ activated astrocytes. Substantial damage to axons was found in these lesions as well. The clear association of demyelination with macrophage infiltration indicates a prominent pathogenic role of innate immune mechanisms at this stage. Around such lesions a rim of activated astrocytes can sometimes be found (Laman et al., 1998). Similar to our own studies ('t Hart et al., 1998), the study by Maggi et al. (2014) revealed a substantial number of lesions without strong inflammatory activity or signs of remyelination and with moderate axonal damage, which they named late subacute lesions. Interestingly, on T2W MR images these latter lesions usually have a smaller size than the acute and subacute lesions, which may be due to the resorption of tissue edema and/or repair ('t Hart and Massacesi, 2009; Maggi et al., 2017).

The main message from these studies is that perivenular inflammation, i.e., lymphocyte cuffs and microglia activation, precedes the formation of lesions. Lesions develop by centrifugal expansion from non-demyelinated nodules of lymphoid and myeloid cells around a leaky blood vessel via which they have infiltrated the CNS parenchyma. Although these observations clearly support a primary inflammatory ontogeny of MS lesions, they do not answer the question of where autoimmunity is induced.

## 12 Targeted dietary intervention in the marmoset EAE model: was Metchnikoff right?

Research discussed thus far has led to the conclusion that the heart of the marmoset EAE model is formed by pathogenic functions that B cells acquire via LCV infection: protection of the vulnerable MOG40-48 epitope against fast degradation, cross-presentation of the epitope via Caja-E to effector memory CTLs and secretion of post-translationally modified MOG34-56 as toxic aggregates, either packaged or not packaged in vesicles. This concept may provide a plausible mechanistic explanation for the association of EBV infection with MS susceptibility.

The clear core pathogenic role of LCV-infected B cells raises the question of whether other environmental factors that influence the disease expression in the model may exert their effect via LCV-infected B cells. A serendipitous finding showed that this may be the case for (a) component(s) of a new dietary supplement that was introduced into the marmoset colony at BPRC in January 2014. Recent literature is flooded with publications about the role of a gut–immune–brain axis in the initiation and course of EAE (Joscelyn and Kasper, 2014; Shahi et al., 2017; Wekerle, 2017). At this point the marmoset EAE model has also yielded some intriguing new insights, which are summarized in the following paragraphs.

A direct consequence of the high level of refinement of the marmoset EAE model has been the loss of robustness of the model, which is probably due to higher sensitivity for the variable influence of genetic and environmental factors. This has led to increased variation in the response to the immunizing antigen or to an experimental treatment (e.g., anti-CD127 mAb), including nonresponders, and the potential loss of statistical power ('t Hart, 2016a). In the course of 4 years (2014–2018) we were facing an unexplained sudden decrement in the disease incidence of the atypical EAE models induced with MOG34-56/IFA (from 90 % to 65 %) or rhMOG/IFA (from 100 % to 60 %) (Fig. 15a). The reduced EAE susceptibility coincided with a sharp decrease in the number of marmosets in the BPRC colony testing positive for CalHV3 (Fig. 15b). It has taken us considerable time to find out that the sudden drop of EAE susceptibility coincided with the modification of a dietary supplement, which was introduced in January 2014. The modification was rather modest, namely from a water-based to a yogurt-based formulation, which also contained the double dose of B vitamins. Nevertheless, the effects on the model were rather dramatic (Kap et al., 2018a).

These enigmatic observations prompted a controlled study in marmoset twins, which had been fed the new dietary supplement from weaning onwards. For the experiment, one sibling of each twin was reverted to the old water-based supplement (WBS), starting 8 weeks prior to EAE induction, while the other sibling remained on the new yogurt-based supplement (YBS). In brief, the EAE incidence in the old WBS and new YBS groups differed, albeit not significantly (8/8 in the old diet group, 6/8 in the new diet group) (Kap et al., 2018a). In addition, we observed a significant reduction of spinal cord demyelination, markedly altered expression of genes involved in apoptosis and myelination in the brain, a marked reduction of CalHV3 expression and alteration of cellular immune parameters, which in previous studies have been implicated in the EAE pathogenesis. As expected, the dietary modification was reflected by a marked alteration of gut microbiota composition. However, diversification of gut microbiota between the WBS and YBS groups became first detectable only 3 weeks after rhMOG/IFA inoculation.

It is unclear at this moment which components of the YBS were responsible for the effects on the model. One candidate is the yogurt. The health benefits of sour milk have been known since biblical times, but a deeper mechanistic understanding comes from the work of the 1908 Nobel Prize winner for physiology or medicine, Ilya Metchnikoff. He proposed that health can be enhanced and aging postponed through manipulation of the gut microbiota with host-friendly bacteria present in fermented milk (Mackowiak, 2013). This is exactly what we found in the twin study (Kap et al., 2018a). We observed a positive effect of the YBS on the proportion of bifidobacteria in the marmoset stool. According to another theory, yogurt is a dietary source of tryptophan, which can be metabolized by the marmoset gut microbiota, the bifidobacteria in particular. Tryptophan can be converted by probiotic gut microbiota into high-affinity ligands of the aryl-hydrocarbon receptor (AHR), such as kynurenine (O'Mahony et al., 2015). Ligand-activated AHR counteracts the increased expression of genes relevant for B-cell growth and function, such as early B-cell factor 1 (EBF1), in EBV-infected B cells (Li et al., 2017). Upregulation of EBF1 induced by EBNA1 is associated with EBNA1's role in maintaining the viability of infected B cells and keeping the EBV genome intact during latent infection (Tempera et al., 2015). This suggests that the yogurt intake in combination with higher levels of bifidobacteria in the gut may lead to the production of factors mediating AHR activation, which might have caused the reduced survival of CalHV3-infected B cells.

The translational relevance of these findings for the MS patient may be that the pathogen-educated immune system of adult outbred marmosets is susceptible to dietary modification in such a way that immune responses against MOG released from a primary lesion in the CNS are reduced. Further work should reveal which components in the YBS diet are responsible for this effect.

## 13 Preclinical efficacy assessment of candidate treatments

One of the strongest arguments for developing nonhuman primate EAE models of MS is that they provide relevant systems for translational research into the pathogenesis and treatment of the disease. Relevant features in this line of research are that many biologicals developed for immunosuppressive or immunomodulatory therapy in MS cross-react between marmosets and humans and that marmosets and humans share a high level of immunological similarity. Indeed, reports from others (Maggi et al., 2017) and us ('t Hart et al., 2015) documented the remarkable similarities in the afferent and effector arms between marmoset EAE and MS.

The diagram in Fig. 1 shows that the integration of exploratory research of pathogenic mechanisms and applied research of new therapies creates a powerful research strategy. The integration of forward and reverse translation creates an iterative scientific process that potentially provides deep insight into the critical steps in the pathogenic process and delivers better therapies. We have used this iterative strategy in the marmoset EAE model. More specifically, we tested therapies that worked or did not work in the clinic for eliminating pathogenic mechanisms in the EAE model that are irrelevant for the human disease. The ultimate aim of this strategy was to create the most relevant preclinical model for MS ('t Hart, 2015, 2017b).

**Figure 16:**
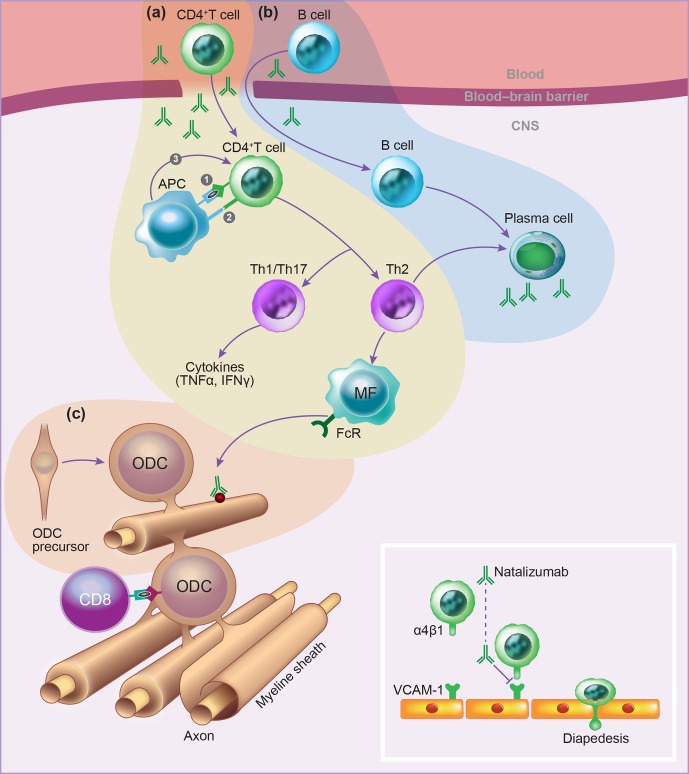
*Schematic overview of immune processes in the EAE model.* **(a)** Autoreactive CD4+ T cells that have been activated in peripheral lymphoid organs by the injection of antigen-adjuvant emulsion infiltrate the CNS via passage through the blood–brain barrier. This passage is mediated by interaction adhesion molecules, such as the α4β1-integrin VLA-4 with ICAM-1 on blood–brain barrier endothelial cells (see insert). By local cognate interactions with local antigen-presenting cells, including dendritic cells, macrophages and microglia, the T cells elicit a cascade of pathophysiological reactions leading to inflammation and demyelination. **(b)** A second infiltrating lymphocyte type is the B cell, which secrete antibodies that, via binding to myelin sheaths and oligodendrocytes, elicit macrophage-mediated and complement-dependent cytotoxicity (ADCC and CDC, respectively). However, this classical role of B cells requires adjustment, because B cells have a much more elaborate pathogenic role. **(c)** The target of the autoimmune process is the axon–myelin unit, which comprises axons, the enwrapping myelin sheaths and the myelin-forming oligodendrocytes. In the healthy CNS, damaged oligodendrocytes can be replaced by infiltrating oligodendrocyte precursor cells (OPCs), but this repair capacity seems impaired in MS.

Figure 16 gives a graphical presentation of relevant factors in the pathogenic process inside the CNS, each of which could be a target for therapeutic intervention. Several of these targets have been validated in the marmoset EAE model:

- It is believed that the immunopathogenic process within the CNS starts with the infiltration of peripherally activated CD4+ T cells with an inflammatory phenotype (a). One type of therapy targets the attachment of the T cells to endothelial cells of the blood–brain barrier, which is mediated by adhesion molecules. An example of a drug approved for clinical use is Natalizumab, a monoclonal antibody directed against α4β1 integrin (VLA-4), which mediated autoreactive T cell infiltration into the CNS in mouse EAE studies (Steinman, 2005) (see insert).
- T cells that have achieved transmigration of the blood–brain barrier need to engage in cognate interaction with local antigen-presenting cells (APCs) for unfolding their complete pathogenic activity. This interaction comprises three signals: signal 1, the formation of a trimolecular complex composed of a peptide presented by MHC molecules and the antigen receptor of T cells; signal 2, the interaction of co-stimulatory molecules expressed by T cells and APCs, such as CD28 with CD80/86 or CD40 ligand with CD40; and signal 3, cytokines that steer functional polarization of the activated T cells, such as IL12, IL-23 and IL-7. A frequently tested approach in the broad spectrum of immune-based inflammatory disorders is the functional ablation of inflammatory T cells by blocking signal 2.
- The infiltration of CD4+ T cells is followed by a second wave of infiltrating immune cells, in particular B cells (b) and macrophages. Macrophages are a dominant cell type in inflammatory active lesions. We actually found that the MRI markers of inflammation in lesions correlate reasonably well with macrophage infiltration in marmoset EAE (Blezer et al., 2007). Mounting evidence indicates that B cells have a multifaceted pathogenic role within the CNS, although there is only solid evidence for production of autoantibodies capable of binding myelin (Genain et al., 1999).
- Mounting evidence indicates that MS patients have an inherently deficient repair capacity for myelin damage by recruitment of oligodendrocyte precursor cells (c) into lesions, where they are supposed to differentiate into mature oligodendrocytes which are capable of producing new myelin sheaths.
  The demyelination process focusses on the oligodendrocyte/myelin–axon units via two mechanisms, namely cytotoxic damage to myelin sheaths via complement (CDC) or macrophages (ADCC) and T-cell-mediated cytotoxicity towards oligodendrocytes.

Below follows an overview of the types of therapy that were tested in the marmoset EAE model.

*Category 1: therapies targeting T cells.* Rodent EAE models show a central pathogenic role of two types of pro-inflammatory CD4+ T helper (Th) cells, which can be distinguished on the basis of their cytokine signature: namely IFNγ+ Th1 cells and IL-17A+ Th17 cells (Gran et al., 2004; Damsker et al., 2010). The differentiation of these functional subtypes from precursor Th0 cells is directed by cytokines produced by the APCs, i.e., IL-12 for Th1 and IL-23 for Th17 (Hunter, 2005). There is ample evidence in the literature that functional or physical elimination of Th1 and/or Th17 cells mitigates the incidence and/or course of EAE in SPF-rodent models.

*IL-12/IL-23.* The CD4+ Th cell-centered concept of MS stimulated the development of a fully human mAb (ustekinumab) against the shared p40 subunit of IL-12 dimer (p35/p40) and IL-23 dimer (p19/p40). However, contrary to expectation, the antibody failed to show relevant activity in RRMS (Segal et al., 2008). Control tests showed that the mAb cross-reacts with marmoset IL-12p40. We have assessed the clinical efficacy of the mAb in two different treatment modes, namely starting treatment just before the immunization (prophylactic) or once brain lesions of sufficient size could be detected and quantified with MRI (therapeutic). In brief, in the prophylactic experiment (n=2×5) EAE was induced with myelin/CFA. All monkeys treated with a placebo preparation (phosphate buffered saline; PBS) developed clinically evident EAE with complete EAE pathology. In the prophylactic experiment EAE was induced with MS myelin/CFA. In all five monkeys treated with the ustekinumab mAb we observed complete suppression of clinical and pathological aspects of EAE (Brok et al., 2002). In the therapeutic experiment (n=2×5) the time of appearance of brain lesions, recorded with MRI, differed between individual animals. All monkeys developed clinical EAE as well as severe pathology. It was observed that MRI parameters for lesion activity (post Gado-T1, T2, lesion surface enlargement) measured after infusion of the mAb did not further change, indicating that pathogenic processes inside the CNS were stopped for the remaining time of the experiment. Nevertheless, the onset time of clinical signs was only delayed for a few weeks ('t Hart et al., 2005c).

In conclusion, the two experiments show that the mAb had a strong beneficial effect when treatment was started early in the disease, while the treatment was less effective once the disease was ongoing. Moreover, we observed a dissociation between the strong effect of the mAb on white matter pathology and the modest, albeit statistically significant, effect on the clinical signs. An explanation for this clinical–pathological paradox may be the existence of two immunopathogenic pathways, an initiation pathway driven by IFNγ+ CD4+ T cells and a progression pathway driven by IL-17+ CD8+ T cells ('t Hart et al., 2011).

*IFN*γ. In a second experiment, we tested the effect of human IFNγ in the MOG34-56/IFA model (Jagessar et al., 2012a). In murine EAE, promising effects of this treatment have been obtained (Sanvito et al., 2010). The cytokine was administered from day 0–25 (prophylactic) or day 65–81 (therapeutic). We observed that the early treatment caused a moderate, albeit statistically not significant, delay of disease onset (from day 98±36 in the control group to day 118±40 in the cytokine-treated group). The late treatment had a more modest effect on EAE onset (from day 98±36 in the control group to day 105±50 in the cytokine-treated group). Moreover, we observed a clear effect of the early treatment on humoral and cellular autoimmune parameters.

In conclusion, these observations argue against an important pathogenic role of Th1 cells in the marmoset EAE model.

*IL-17A.* In a third experiment, we tested the effect of a humanized IgG4κ mAb against human IL-17A in the rhMOG/CFA model. Biocore tests showed that the reactivity with marmoset IL-17A was about 2-fold lower than with human IL-17A, whereas in a bioassay the activity was 4-fold lower (Kap et al., 2011b). The mAb was tested in two doses in the marmoset EAE model, 3 or 30 mg kg${}^{-1}$, and the effect was compared with PBS as placebo. The data showed a trend towards delayed EAE onset in the low-dose group (mean day 69.3; 48–113) compared with the control group (mean day 57.1; 39–91) and the high-dose group (mean day 60; 51–69). Moreover, the EAE progression time from EAE score 2 (ataxia) to score 3 (paraplegia) was faster in the control group (mean 10.8 d; 3–20) than in the low (mean 12.9 d; range 5–28) and high (mean 13.7; 6–31) mAb dose groups. These differences were statistically not significant. Also, the pathological examination showed no significant effect of the mAb.

In conclusion, we found that treatment with anti-IL-17A antibody induced a moderate delay of clinical EAE in marmosets, but EAE development was not completely abrogated. This finding hints at a pathogenic role for IL-17A in the marmoset EAE model and maybe in MS, but IL-17A may not be the only key pathogenic cytokine.

*IL-7 receptor/CD127.* IL-7 is a cytokine produced by stroma cells that has a broad activity range. In the bone marrow, IL-7 stimulates the differentiation of hemopoietic stem cells into lymphoid progenitor cells. Moreover, the cytokine stimulates proliferation and differentiation of T cells, B cells and NK cells. We have tested a chimeric IgG mAb raised against marmoset IL-7 in the MOG34-56/IFA model (Dunham et al., 2016). Treatment of seven twins with mAb or PBS intravenously administered was started at 21 d after the first immunization. Although the mAb blocked CD127 function in all monkeys, as assessed by IL-7-induced STAT5 phosphorylation, we observed a beneficial effect on the disease course only in twins with fast-progressing disease. This remarkable dichotomous effect indicates that even in the highly refined MOG34-56/IFA model disease heterogeneity can be observed.

In conclusion, The IL-7 pathway seems a relevant target of therapy for a subset of MS patients.

*Overall conclusion of this part.* These experiments seem to confirm observations in MS clinical trials, namely that therapies targeting only CD4+ T cells are only moderately effective.

*Therapies targeting B cells.* The remarkable results of the anti-CD20 mAb rituximab in RRMS (Hauser et al., 2008) created a paradigm shift in MS. While B cells were for a long time viewed only as producers of myelin opsonizing antibodies, which induce demyelination via complement- (CDC) or macrophage-dependent (ADCC) cytotoxicity reactions, they have now gained a central position in the MS pathogenic process (von Budingen et al., 2015). These findings prompted us to test the second generation anti-CD20 mAb HuMab7D8, which is a clonal variant of the human mAb ofatumumab with confirmed cross-reactivity with marmoset B cells in the EAE model. We made a number of noticeable observations.

*CD20.* We tested the efficacy of HuMab7D8 in the rhMOG/CFA model, starting weekly treatment at 21 d postimmunization. The antibody induced almost complete and persistent depletion of CD20+ B cells from blood and lymphoid organs as well as from the CNS (Kap et al., 2010). The treatment led to almost complete suppression of clinical signs of EAE as well of CNS pathology in WM and cGM (Kap et al., 2010, 2011a). Analysis of autoimmune reactions revealed suppression of antibody production as well as T-cell responses against the immunizing rhMOG protein (Kap et al., 2010). Intriguingly, the emptied B cell areas in secondary lymphoid organs of mAb-treated monkeys were replenished by T cells expressing CD127 (IL-7 receptor) and CCR7 (Kap et al., 2014). As discussed above, treatment with anti-CD127 mAb induced substantially delayed EAE onset in fast disease progressor monkeys (Dunham et al., 2016), which was a hallmark of the EAE progression pathway (Kap et al., 2008). These findings combined indicate that treatment with anti-CD20 mAb prohibits the release of activated autoaggressive T cells from the secondary lymphoid organs.

One of the presumed roles of B cells in MS is antigen presentation to autoaggressive T cells (von Budingen et al., 2015). This was tested in the MOG34-56/IFA model, in which pathology and disease is induced by the action of autoaggressive T cells, without the support of myelin-opsonizing autoantibodies. Also in this model, we observed profound suppression of EAE symptoms and pathology, as well as suppression of humoral and cellular autoimmune parameters (Jagessar et al., 2012b).

*BlyS and APRIL.* An alternative method for B cell depletion is the capture of cytokines that B cells need for survival and differentiation, i.e., BlyS/BAFF or APRIL (Dillon et al., 2006). This prompted us to test two mAbs in the rhMOG/CFA model, namely anti-human BlyS mAb (belimumab) and an anti-human APRIL mAb. Treatment was again started at 21 d after the immunization. Both antibodies caused only a moderate, albeit statistically significant, delay of the EAE onset (Jagessar et al., 2012c). Note, examination of the secondary lymphoid organs did not reveal retention of activated autoaggressive T cells (Kap et al., 2014).

*CD40.* The treatments with anti-BlyS/APRIL and anti-CD127 mAbs hinted at a special role of B cells expressing high levels of CD40 in the EAE model (see above). This conclusion is supported by data from earlier studies in which we tested murine, chimeric and humanized variants of the human CD40 blocking mAb 5D12 in the EAE model. In brief, we observed in a marmoset EAE model induced with human myelin/CFA that treatment with a mouse anti-human CD40 mAb (5D12) delays the onset of clinical signs (Laman et al., 2002). In the same study, we observed that the mAb gains access to inflammatory active lesions. To assess whether treatment efficacy might be limited by the formation of antidrug antibodies we gave a single intravenous injection of the murine mAb and tested serum antibody levels at several time points thereafter. Indeed, free test substance levels were substantially lower in mAb-treatment cases than in a naïve monkey.

Based on these encouraging findings, a chimeric mouse–human IgG4 mAb was developed (ch5D12), which was tested in the EAE model induced with rhMOG/CFA (Boon et al., 2002). Also in this experiment, we observed profound suppression of EAE clinical signs and partial suppression of brain and spinal cord pathology. As was expected, the induction of anti-rhMOG IgG antibodies was suppressed as well.

*Overall conclusion.* These immunotherapy findings support a core pathogenic role of CD20+CD40+ B cells in the marmoset EAE model.

*Cell-based regenerative therapies.* One of the great challenges in MS research is to develop treatments with which CNS damage can be repaired once the (immuno)pathogenic process has been stopped. Much is expected from treatment with stem cells (SCs), which are now available in different flavors, such as hemopoietic SCs, neural stem/precursor cells (NPCs), mesenchymal SCs or induced pluripotent stem cells (iPSs) (Martino et al., 2010; Di Ruscio et al., 2015). We have tested two types of stem cells in the marmoset EAE model, namely human NPC, grown as homogenous cell line, and iPS cells derived from reprogrammed human skin fibroblasts, differentiated into oligodendrocyte precursor cells (OPCs).

- *NPC therapy.* This form of therapy is based on the administration of self-renewing, multipotent cells isolated from the CNS. For our study, we used human eGFP-transduced NPC, grown as a homogenous cell line, to enable the detection of injected cells in postmortem brain tissue (Pluchino et al., 2009). The cells were administered to rhMOG/CFA-immunized monkeys either intra-CSF via injection into the cisterna magna or into the blood stream via the tail vein. We injected a low dose of cyclosporine A (CsA; 10 mg kg${}^{-1}$) to prevent immediate rejection of the xenogeneic (human) NPCs. Interestingly, the administration of CsA transformed the natural, rapidly progressing disease course into a chronic relapsing course. Significant clinical amelioration was observed both with the intravenous and the intra-CSF treatments, over an observation period of 90 d. Even after this long time interval, eGFP+ve cells could still be detected in the marmoset CNS. However, we did not observe signs of newly formed neurons or glia cells.
- *OPC therapy.* The scientific impact of the Nobel Prize-winning new technology for reprogramming of committed somatic cells into pluripotent stem cells from which other cell lineages can be generated has been immense (Takahashi and Yamanaka, 2006). We have used this exciting technology to generate OPCs from reprogrammed human skin fibroblasts (Thiruvalluvan et al., 2016). The potency of these (GFP-labeled) OPCs to remyelinate axons that were denuded by autoimmune demyelination was tested in the MOG34-56/IFA marmoset EAE model, The OPC graft was injected into the brain at some distance from an MRI-detectable (T2) lesion. Like in the previous experiment, low dose CsA (10 mg kg${}^{-1}$) was injected to prevent xenorejection of the human OPC. Our study showed that the injected OPC migrated from the injection site to the lesion, where they differentiated into mature oligodendrocytes, which made contact with a denuded axon and started forming a new myelin sheath (Thiruvalluvan et al., 2016).

*Conclusion.* the marmoset EAE model has proven its value for the efficacy assessment of innovative treatments. Relevant features for this line of research are (1) the immunological similarity between marmosets and humans and (2) the neuroanatomical similarity of marmoset and humans.

## 14 Ethical considerations on nonhuman primates as a model of human disease

### 14.1 3 or 4 R's?

The leading principles in preclinical research with living animals have been formulated by Russell and Burch as the “3 R's”: *replacement*, *reduction* and *refinement* (Russell and Burch, 1959). Strict adherence to these principles is now common practice, especially in research with sentient animals, such as NHPs. However, it is rather remarkable that an obviously important fourth R is lacking, namely the (clinical) relevance of an animal model. In the following, these four R's will be briefly discussed as well as why difficulties encountered when harmonizing them have made the generation of a clinically relevant EAE model an acrobatic balancing act ('t Hart, 2016a).

The replacement principle encourages scientists to use methods in their research that avoid or replace the use of living animals. Although in vitro models with cells or tissues and in silico models of human pathology are increasingly used in preclinical research, it is generally felt that the complexity of a pathological process in relation with other physiological body systems is only displayed in living animals. In that case the lowest animal species from which relevant information can be obtained should be selected. This consideration underlies the increasing use of evolutionary distant model systems, such as zebrafish (*Brachydanio rerio*), Drosophila flies and *Caenorhabditis elegans* worms. However, although important insights into novel disease mechanisms can be obtained from these models, it is felt that model systems more closely related to the patient are needed for the integration of all information into a coherent pathogenic concept that can be translated into new therapies.

The reduction principle encourages scientists to use research methods that provide either comparable levels of information from fewer animals or more information from the same number of animals. We achieved the latter goal by the application of nuclear magnetic resonance (NMR) imaging techniques ('t Hart et al., 2004), but compliance with the former condition appeared to be more problematic. Reviewers of projects and publications often limit the interpretation of the reduction principle only to the minimal number of animals that should be used for a “usable” result. Power calculations are applied to determine the minimal size of experimental and control groups, so that the effect of an experimental variable can be statistically tested. Critical variables in power calculations are the disease incidence, the anticipated treatment effect and the expected variation in read-out parameters, such as the time to clinically evident disease or the disease severity. The outbred nature of NHPs and their conventional housing conditions imply a variable influence of genetic background and environmental factors, ironically being dominant autoimmune disease risk factors in the human population. The inevitable consequence of variation is that experimental groups may contain clinically low responders or nonresponders, which reduces disease incidence and thus requires larger group size for statistical power. In rodent disease models this dilemma is bypassed by using well-established genetically homogeneous (inbred) SPF-bred strains and the usage of potent adjuvants to obtain high disease incidence and a synchronous disease course. However, a recent study raised questions on the relevance of immunologically immature SPF mice as models of complex human diseases (Beura et al., 2016). Moreover, the usage of strong bacterial adjuvants clearly conflicts with the refinement and relevance principles (see below).

The refinement principle encourages scientists to employ research methods that minimize the discomfort from experimental procedures to the animals. A major concern with respect to the EAE model is that for reproducible disease induction strong adjuvants need to be used to pepper the immunogenic potency of self-antigens by disrupting the regulatory mechanisms that keep autoaggressive T and B cells under control (Matzinger, 1994). The most frequently used is complete Freund's adjuvant (CFA), which is an emulsion of heat-killed mycobacteria (*M. tuberculosis* or *butyricum*) in mineral oil. The mineral oil, also known as incomplete Freund's adjuvant (IFA), forms a depot for the finely dispersed antigen solution in aqueous buffer; the mycobacteria provide danger signals needed for “awakening” of the tolerized T and B cells. However, CFA is notorious for its seriously detrimental side effects, in particular the induction of severe ulcerative skin lesions at the injection sites, which are clearly caused by the mycobacteria. Usage of CFA in nonhuman primates is therefore discouraged. As discussed above, in the marmoset EAE model IFA can be used, which implies a major reduction of discomfort to immunized animals compared to animals immunized with CFA.

The forgotten fourth R of relevance: the usage of CFA also introduces a mechanistic bias in the EAE model as immune responses against antigens formulated with CFA are skewed towards a pro-inflammatory profile that is dominated by CD4+ T cells (Billiau and Matthys, 2001). The poor translation record of experimental therapies targeting CD4+ T cells from EAE to MS indicates that this lymphocyte subset may be less relevant for the human disease than in the animal model (Hohlfeld et al., 2015). However, this does not preclude a pathogenic role of CD4+ T cells early in the disease process, i.e., before the diagnosis has been made.

Compliance of the marmoset EAE model with the fourth R has been achieved via an iterative strategy depicted in Fig. 13. In brief, research in the exploratory arm aimed at maximum refinement of the original EAE model, which was induced by immunization with myelin isolated from the brain of an MS patient formulated with the bacterial adjuvant CFA ('t Hart et al., 1998). The stepwise refinement of this complex model towards the minimally needed components yielded a highly refined model that displays essential pathological aspects of MS ('t Hart et al., 2015) (Fig. 7). In the applied arm, the consecutive steps of the refinement process were validated with clinically relevant therapeutic mAbs. The *in depth* characterization of this atypical EAE model is still ongoing, but preliminary data show that the MHC-E-restricted CTL that mediate the development of chronic EAE in marmosets (Jagessar et al., 2012d) can also be found in MS lesions (Zaguia et al., 2013).

The replacement of CFA for IFA in the translationally relevant atypical EAE models had various important consequences:

1. The discomfort to the animals in experiment was reduced,
2. The immunogenicity of administered biological therapeutics, which limited their activity window, was reduced,
3. The dogma that danger signals are absolutely needed for autoimmunity induction may need to be adjusted,
4. A new pathogenic mechanism was discovered.

Concerning the third issue a caveat may be needed as the injection of antigen/IFA emulsion will certainly cause damage and induce the release of damage-associated molecular patterns, which can relay danger signals to APCs through DAMP receptors (Kono and Rock, 2008). The released DAMPs cause visible skin irritation. However, these signals appear too weak for the elicitation of EAE in genetically susceptible, but immunologically immature, SPF mice (Jagessar et al., 2010).

### 14.2 A conflict among the R's

Compliance of the primate EAE model with the refinement and relevance principles introduced an unforeseen conflict with the reduction principle. It was observed that the replacement of CFA with IFA produced less robust EAE models, which were more prone to variation in the response against immunization as well as in the response to treatment. A likely explanation is that these refined disease models are more sensitive to the variable influences of genetic and environmental factors. An experiment in which we encountered the consequences was reported recently (Dunham et al., 2016). In brief, we tested the efficacy of a mAb raised against the human IL-7 receptor CD127 in a powered two-leg study in marmoset twins (n=7) immunized with MOG peptide 34-56/IFA (Dunham et al., 2016). One sibling of each twin received the therapeutic mAb and the other a placebo preparation. We observed that one twin pair did not develop clinical EAE within the 150 d observation period, while in the six twin pairs that did develop EAE no statistically significant effect was observed at the group level. However, at the individual twin level we observed that with a fast disease evolution in three twins the treatment had a clear clinical effect, while no effect of the mAb was observed in three twins with a slowly evolving disease. The lack of statistical significance at the group level made publication of the data highly problematic.

How should we interpret this experiment? Should further development of the mAb be stopped because a significant effect of the treatment could not be proven, even when the number of animals per group is doubled or tripled? Or might the mAb be clinically relevant for a subset of the patients, namely those with fast disease progression? This is not a theoretical issue as patients variation in the response to treatment (with interferon-β) has been also observed in MS (Axtell et al., 2010).

The prisoner's dilemma here is that when there are no markers for disease progression rate that can be used for preselection of high responder animals, the only way to achieve statistical significance is increasing the group size. This obviously creates a conflict with the reduction principle.

There seems to be no easy solution for the apparent conflict between (statistical) significance and (clinical) relevance for the highly refined EAE models. One solution could be that reviewers of grants and publications on proof of concept studies choose not to apply the normal sample size dogmas developed for homogeneous models, such as those in inbred/SPF mice, to studies in more complex and more heterogeneous disease models in nonhuman primates (Bacchetti et al., 2011, 2012). The central argument is that the value of a study does not increase proportionally with the number of animals added to a test group, while the burden from discomfort on study participants does increment proportionally (Bacchetti et al., 2005).

## 15 Concluding remarks and open questions for further research

A final relevant question is whether insights that we gained through studies of the marmoset EAE model may help explain the disease course depicted in Fig. 4b. I posit the concept that the course of EAE in marmosets is driven by post-translational modifications of the immunodominant myelin component MOG.

*From tolerance to autoimmunity.* We showed that MOG in the healthy brain maintains homeostasis via the interaction of its glycan epitope attached at the asparagine (Asn/N) 31 residue with the C-type lectin receptor DC-SIGN, which is expressed on microglia and dendritic cells in the draining (cervical and lumbar) lymph nodes. Modification of this glycan, which may either have a genetic or a pathological cause (inflammation for example) uncovers the pathogenic role of MOG (Garcia-Vallejo et al., 2014). Studies in the marmoset EAE model show that nonglycosylated rhMOG protein or MOG peptide are strongly immunogenic and encephalitogenic. It is therefore proposed that autoimmunity against MOG is induced when the protein loses its tolerogenic glycan epitope.

*From autoimmunity to relapsing disease.* We identified two epitopes, MOG24-36 (CD4+ T cells) and MOG40-48 (CD8+ T cells). A recent study in mice may help understand why these two epitopes are immunodominant. It was shown that epitopes containing an SP or PP motif are destroyed in the thymus by the serine protease TSSP (Serre et al., 2017). MOG contains three SP motifs: residues 27 and 28, which are located in the CD4 epitope; residues 42 and 43, which are located in the CD8 epitope and residues 123 and 124, which have not been located in a known T cell epitope in marmosets, although it is a T cell epitope in B6 mice (MOG119-128) (Shetty et al., 2014). Thus, the two marmoset T cell epitopes contain an SP motif, which may implicate their destruction in thymic epithelial cells, as a consequence of which T cells against these specificities escape negative selection from the immune repertoire.

A second relevant post-translational modification of myelin induced under inflammatory conditions is the replacement of arginine residues by citrulline, a process indicated as citrullination. Enzymatic citrullination is mediated by peptidyl-arginine deïminase (PAD) enzymes, which exist in five isoforms (PAD1, 2, 3, 4/5 and 6); PAD2 is the most prevalent in the CNS (Vossenaar et al., 2003). Full activity of PADs depends on local [${\mathrm{Ca}}^{2+}$]. The replacement of positively charged arginine residues in peptides/proteins by neutrally charged citrulline can affect the conformation and function of proteins. It has been shown that the proportion of citrullinated MBP is substantially higher in the MS brain than in healthy tissue (45 % vs. 18 %), while in Marburg's acute MS as much as 90 % of MBP is citrullinated (Moscarello et al., 1994; Wood et al., 2008). In the early phase of MS, and at a young age of the patient, only a minor fraction of MOG is citrullinated. It can thus be envisaged that in the early phase of MS the immune system sees only a low citrullination grade of myelin antigens.

Data obtained in the rhMOG/IFA model show that the immune response against the noncitrullinated protein is restricted to pro-inflammatory CD4+ T cells against MOG24-36 and antibodies against the conformational epitope located at the apical end of monomeric MOG. The combined activity of these factors mediates the initial autoimmune attack on the myelin sheaths in the white matter. As oligodendrocytes are spared, remyelination can occur. T-cell reactivity against MOG34-56 is not induced, which is probably due to the destructive processing of the peptide by the endolysosomal serine protease cathepsin G. Conceptually, once the autoimmune inflammation is strong enough to exceed a clinical threshold, neurological deficits are detectable (relapse), which are antagonized by counter-regulatory mechanisms that suppress T cell inflammation, such as Treg cells, corticosteroids and anti-inflammatory cytokines (TGFβ, IL-10). We posit that the recurrent activation and suppression of this autoimmune–inflammatory pathology underlies the relapsing–remitting course. An important finding has been the discovery that engagement of T cells against the MOG34-56 peptide strongly accelerates EAE development (Kap et al., 2008). Intriguingly, blockade of the IL-7 receptor with an anti-human CD127 MoAb abrogated accelerated EAE development (Dunham et al., 2016). This resembles the situation in MS where IL-7-responsive T cells specific for MOG34-56 were identified as one of six specificities distinguishing MS patients from healthy controls (Bielekova et al., 2004).

In silico 3-D modeling of human MOG (Fig. 10) shows that the sequence 34–56 is exposed at the surface of the molecule. The thee Arg residues are highlighted, showing that the Arg${}^{46}$ and Arg${}^{52}$ residues are freely accessible, while the Arg${}^{41}$ residue is somewhat buried. Citrullination of the Arg46 residue, which is relatively more surface exposed than Arg${}^{41}$ is sufficient for the association of the peptide with autophagosomes in APCs. The ensuing protection of the peptide against fast degradation warrants availability of the epitope for cross-presentation via Caja-E to CD8+CD56+ CTL. This CTL activation requires the involvement of LCV-infected B cells, which possess mechanisms (autophagy, citrullination) to protect the MOG40-48 epitope against fast degradation by cathepsin G and cross-presentation. In this phase of the disease both CD4+ and CD8+ T-cell responses can be measured. Via the CTL a novel pathogenic pathway is activated, which mediates an attack on the cortical grey matter.

*From relapsing to progressive disease.* Literature data suggest that with the progression of time (and disease severity) the proportion of myelin protein that is citrullinated increases (Moscarello et al., 1994). By the citrullination, T-cell recognition of the antigen is disturbed, while the tendency to spontaneously form amyloid-type aggregates increases. Citrullination of the Arg${}^{52}$ residue appears to be particularly important for aggregation as this modification stabilizes the β sheets within the encephalitogenic MOG34-56 peptide. This feature activates a third pathogenic mechanism in which LCV-infected B cells have a central, albeit non-immunological, role. The available data, albeit still scarce, suggest that EBV-infected B cells have a role in aggregate formation and in the packaging of aggregates in vesicles. The aggregates seem to induce apoptosis in cocultures of EBV-infected B cells and MOG-reactive T cells. However, it still remains to be proven whether aggregates secreted by the EBV-infected B cells are toxic for CNS cells. Nevertheless, we believe that the data provide a potentially relevant concept for progressive MS.

*Open questions.* The marmoset EAE model is now regarded by many as the translationally most relevant animal model of MS. Important aspects of the model are the clear pathogenic role of B cells infected with the lymphocryptovirus CalHV3. However, it is still an open question of whether the mechanisms uncovered in the model are relevant for the human disease.

The EAE model in marmosets is pathologically characterized by lesions in the white and grey matter of the brain and the spinal cord. The model also revealed that lesions in white and grey matter are induced via distinct pathogenic mechanisms. Also, the question of whether the mechanisms that we have explored are translatable to MS is still open.

An intriguing new finding is that toxic amyloid-like aggregates are formed in the interaction between citrullinated MOG34-56 peptide with EBV-infected B cells. It is still an open question as to whether these aggregates are secreted by the B cells, in which form this occurs and whether the aggregates are toxic towards neurons and glia cells.

## Data Availability

The author of this monography has retired and has no access to primary data. Primary data on the pathological characterization of the model can be found in reference 't Hart et al. (1998). Primary immunology data of the model can be found in references Brok et al. (2000), Kap et al. (2008) and Jagessar et al. (2010, 2012d). Papers presenting primary data on the pathogenic role of EBV-infected B cells are Jagessar et al. (2013a), (2016) and Morandi (2017b) and Araman et al. (2019).
